# Transcriptomes of Clusterin- and S100B-transfected neuronal cells elucidate protective mechanisms against hypoxia and oxidative stress in the hooded seal (*Cystophora cristata*) brain

**DOI:** 10.1186/s12868-022-00744-6

**Published:** 2022-10-15

**Authors:** Gerrit A. Martens, Cornelia Geßner, Carina Osterhof, Thomas Hankeln, Thorsten Burmester

**Affiliations:** 1 Institute of Animal Cell and Systems Biology, Biocenter Grindel, University of Hamburg, 20146 Hamburg, Germany; 2 Institute of Organismic and Molecular Evolution, Molecular Genetics & Genome Analysis, Johannes Gutenberg University Mainz, 55128 Mainz, Germany

**Keywords:** Clusterin, S100B, Hypoxia, Oxidative stress, Transcriptome, Neurons, Brain, Hooded seal, Marine mammals

## Abstract

**Background:**

The hooded seal (*Cystophora cristata*) exhibits impressive diving skills and can tolerate extended durations of asphyxia, hypoxia and oxidative stress, without suffering from irreversible neuronal damage. Thus, when exposed to hypoxia in vitro, neurons of fresh cortical and hippocampal tissue from hooded seals maintained their membrane potential 4–5 times longer than neurons of mice. We aimed to identify the molecular mechanisms underlying the intrinsic neuronal hypoxia tolerance. Previous comparative transcriptomics of the visual cortex have revealed that S100B and clusterin (apolipoprotein J), two stress proteins that are involved in neurological disorders characterized by hypoxic conditions, have a remarkably high expression in hooded seals compared to ferrets. When overexpressed in murine neuronal cells (HN33), S100B and clusterin had neuroprotective effects when cells were exposed to hypoxia. However, their specific roles in hypoxia have remained largely unknown.

**Methods:**

In order to shed light on potential molecular pathways or interaction partners, we exposed HN33 cells transfected with either S100B, soluble clusterin (sCLU) or nuclear clusterin (nCLU) to normoxia, hypoxia and oxidative stress for 24 h. We then determined cell viability and compared the transcriptomes of transfected cells to control cells. Potential pathways and upstream regulators were identified via Gene Ontology (GO) and Ingenuity Pathway Analysis (IPA).

**Results:**

HN33 cells transfected with sCLU and S100B demonstrated improved glycolytic capacity and reduced aerobic respiration at normoxic conditions. Additionally, sCLU appeared to enhance pathways for cellular homeostasis to counteract stress-induced aggregation of proteins. S100B-transfected cells sustained lowered energy-intensive synaptic signaling. In response to hypoxia, hypoxia-inducible factor (HIF) pathways were considerably elevated in nCLU- and sCLU-transfected cells. In a previous study, S100B and sCLU decreased the amount of reactive oxygen species and lipid peroxidation in HN33 cells in response to oxidative stress, but in the present study, these functional effects were not mirrored in gene expression changes.

**Conclusions:**

sCLU and S100B overexpression increased neuronal survival by decreasing aerobic metabolism and synaptic signaling in advance to hypoxia and oxidative stress conditions, possibly to reduce energy expenditure and the build-up of deleterious reactive oxygen species (ROS). Thus, a high expression of CLU isoforms and S100B is likely beneficial during hypoxic conditions.

**Supplementary Information:**

The online version contains supplementary material available at 10.1186/s12868-022-00744-6.

## Introduction

The hooded seal (*Cystophora cristata*) is an excellent breath-hold diver, performing dives for up to 1 h, while diving over 1 km deep [24, 98]. Physiological adaptations, such as increased oxygen stores (hemoglobin, myoglobin), a decreased heart rate (bradycardia) and a redirection of blood flow to vital organs (selective peripheral vasoconstriction) have evolved to facilitate this diving lifestyle [6, 7, 23, 81, 82, 94]. However, during repetitive diving bouts oxygen partial pressure may drop dramatically, as shown in other deep-diving seals [69, 84], and would lead to neuronal damage in humans [66]. Neurons from the hooded seal have an intrinsic hypoxia tolerance that cannot be explained by these physiological adaptations. Isolated hooded seal brain slices maintained their membrane potential during hypoxic treatment, while those of mice lost their functional integrity [25]. The molecular basis of this intrinsic hypoxia tolerance is not well understood. In a comparative transcriptomics analysis, the calcium-binding protein S100B and the molecular chaperone clusterin (CLU) emerged as highly expressed in the hooded seal visual cortex, when compared to the ferret (*Mustela putorius furo*). More precisely, S100B expression was 38-fold higher in the hooded seal than in the ferret [20]. CLU exhibited the highest mRNA levels in the hooded seal cortex, with a fourfold increase compared to the ferret [20]. The remarkably enhanced transcription of S100B was confirmed in laser-excised hooded seal neurons, in which S100B was 82-fold more highly expressed than in neurons of mice (*Mus musculus*) [30]. The observed overexpression suggests that both genes might contribute to the hypoxia tolerance of the hooded seal brain [20, 30]. Both, S100B and CLU, are associated with many neurological disorders such as Alzheimer’s disease and Parkinson’s disease in humans that involve hypoxia and oxidative stress [27, 71]. However, their role and molecular mechanisms in these conditions are controversial and ambiguous.

The S100 proteins are appreciably conserved among different species [21, 31], which may indicate crucially conserved biological roles. S100B is a calcium-binding protein that is involved in a broad range of Ca^2+^-dependent pathways [72]. Human and rodent studies demonstrated S100B’s dual role, acting as an intracellular regulator on the one hand and as an extracellular signal substance on the other hand [17]. Intracellular S100B is involved in many processes such as proliferation, differentiation and survival [72]. For instance, in melanoma cells S100B leads to improved tumor survival, preventing p53-mediated apoptosis [64, 65]. Although mainly present in astrocytes [71], S100B was also found to be located in neurons [88], in which it reduced apoptosis and nerve growth factor (NGF)-induced differentiation [2]. S100B may be secreted from astrocytes and neurons in conditions of metabolic stress and other external stimuli [18, 29], where its role depends on its concentration [72]. When released into the extracellular microenvironment, S100B acts as a damage-associated molecular pattern (DAMP) protein through its interaction with the receptor for advanced glycation end products (RAGE) [13, 96]. RAGE is a multi-ligand receptor of the immunoglobulin superfamily which is mainly expressed by neurons and microglia and which mediates inflammatory responses by activating multiple signaling pathways [1, 44]. At nanomolar concentrations, S100B demonstrates neurotrophic effects, promoting neurite extension and neuron survival, modulating long-term potentiation and counteracting neurotoxicants like reactive oxygen species (ROS) [71]. Neuron survival may be facilitated by different RAGE-dependent pathways [17, 42, 59]. By contrast, persistent activation of RAGE by micromolar concentrations of S100B produces increased amounts of ROS, leading to lipid peroxidation and consequently induction of apoptosis [99]. However, studies report varying results at what concentration S100B exerts neurotrophic or neurotoxic effects [17]. In neuronal disorders such as acute brain injury and neurodegenerative diseases, S100B has been found at high levels serving as a biomarker of disease progression [71]. Nevertheless, in proteinopathies like Alzheimer’s and Parkinson’s disease, S100B might also be involved in clearance of detrimental protein aggregates [72].

CLU, a multifunctional glycoprotein, is a constitutively secreted chaperone in its predominant form (soluble CLU, sCLU), but truncated forms localized to the nucleus (nuclear CLU, nCLU) have also been found [38, 45]. The different isoforms of CLU target distinct cellular or subcellular localizations in the rat and human brain, where they demonstrate different functions [38]. The sCLU isoform is translated as a pre-protein of 36–39 kDa, which contains an N-terminal endoplasmic reticulum (ER)-signaling peptide and two nuclear localization sequences. After removal of the signaling peptide, the pre-protein is phosphorylated and glycosylated in the ER and Golgi body. Cleavage of the intermediate glycoprotein results in two subunits linked by disulfide-bonds. The resulting mature antiparallel, heterodimeric glycoprotein (70–75 kDa), commonly referred to as sCLU, is then secreted [8]. In contrast, nCLU is a truncated isoform of 45–50 kDa, lacking the ER signaling peptide and is primarily detected in the cytosol and nucleus [62, 83]. Other CLU forms targeted to the mitochondria have also been described, where they may function as antiapoptotic proteins during stress conditions or facilitate mitochondrial respiration [38, 89, 103]. Although some studies question the relevance and existence of CLU isoforms [93], it has been demonstrated that sCLU and nCLU exhibit distinct functions and that they regulate certain cellular processes in opposite manners [38]. CLU function and expression is regulated by a wide variety of signals including growth and transcription factors, as well as several stress conditions [27]. For instance, during oxidative stress conditions, intracellular CLU promotes cardiomyocyte survival [47]. Due to its stress-increased expression and extracellular chaperone activity CLU has been compared to heat shock proteins [27]. Indeed, CLU might mediate neuroprotection by preventing stress-induced precipitation and aggregation of proteins, by mediating clearance of extracellular misfolded proteins and aggregates, and by promoting their cellular uptake [100]. Additionally, cytosolic CLU might have an important role in intracellular protein homeostasis (proteostasis), by transporting misfolded proteins to the proteasome and/or autophagy for degradation [34, 68, 102]. Similar to S100B, in proteinopathies like Alzheimer’s and Parkinson’s disease, CLU has been found to associate with protein aggregates and might be involved in their clearance [27, 61]. However, in advanced disease stages CLU has been found to promote neurotoxicity [101]. Its concentration in peripheral blood was identified as a potential biomarker for neurodegenerative diseases, like Alzheimer’s disease [4]. Still, CLU’s downstream pathways and molecular mechanisms are not well characterized. Different cell-types, varying levels of model complexity, and conditions that represent distinct physiological situations might lead to differing conclusions [27]. Furthermore, lack of discrimination between different CLU isoforms as well as structural differences between these proteins (e.g., glycosylation levels) may be key in explaining the variability in CLU effects in apoptosis and cell death pathways.

Remarkably, the protective effects of S100B and CLU could be demonstrated in cell culture experiments [31]. Murine neuronal cell cultures (HN33) that were transfected with S100B, sCLU and nCLU demonstrated elevated viability when exposed to hypoxia [31]. Furthermore, overexpression of S100B, sCLU and, to a lesser degree, nCLU, led to a reduction of ROS and lipid peroxidation [31]. While these findings confirmed neuroprotective roles of S100B and CLU in hypoxia and oxidative stress, their molecular mechanisms remained obscure. In order to improve our understanding of the roles of these proteins in cell metabolism, we analysed the transcriptomes of neuronal cell cultures transfected with S100B, nCLU and sCLU and then exposed to normoxia, hypoxia and oxidative stress, with the aim to identify possible molecular targets and pathways of S100B and CLU at different oxygen regimes.

## Results and discussion

### Overexpression of transfected genes in HN33 cell lines

Transfected HN33 cell lines demonstrated stable mRNA overexpression of the S100B, sCLU and nCLU transgenes compared to the endogenous expression levels measured in mock-transfected cells. Overexpression in qPCR experiments ranged from 712-fold for S100B to 9230–9723-fold for nCLU and sCLU, respectively (Additional file 1: Fig. S1). Expression of transfected genes was also observed in the RNA-Seq transcriptome data, when mapped to the mouse reference genome with added transgenic sequences (Additional file 1: Fig. S2).

### Viability of HN33 cell lines

**Fig. 1:**
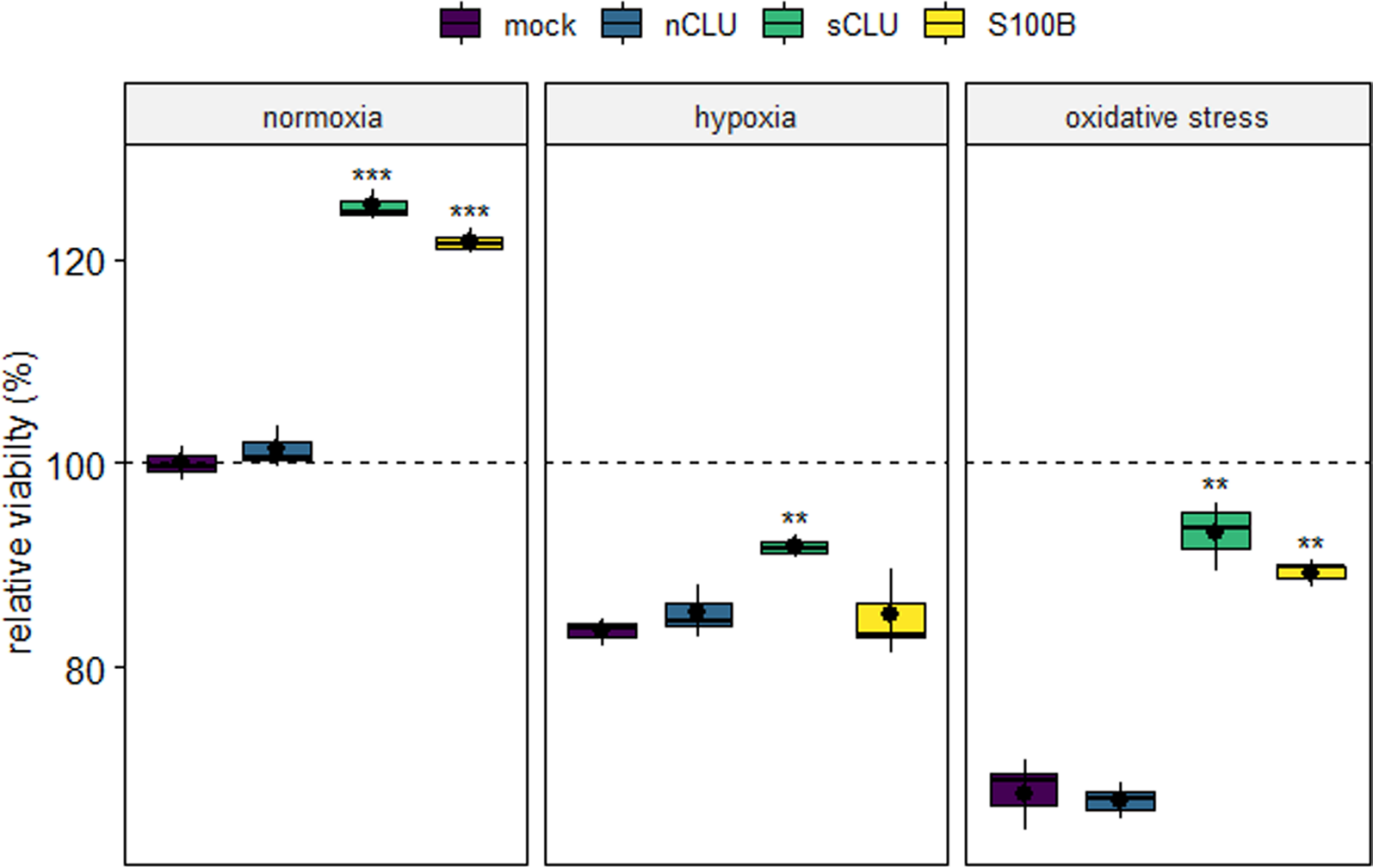
Relative viability (%) of transfected cell lines compared to a mock cell line at the respective stress conditions. After exposure to normoxia, hypoxia and oxidative stress for 24 h, cell viability was measured by CellTiter-Glo® assay. Viability of transfected cells (n = 3) was compared to the mock cell line, at each respective stress condition. The dashed line indicates viability of the mock cell line at normoxia. ****p_FDR_ < 0.0001, ***p_FDR_ < 0.001, **p_FDR_ < 0.01, *p_FDR_ < 0.05

Cell lines exhibited significant differences in ATP amounts, as shown by the CTG cell viability assay after exposure to normoxia, hypoxia and oxidative stress (275 µM H_2_O_2_) for 24 h (Fig. 1). Cells transfected with sCLU and S100B were more viable at normoxic conditions than the mock and nCLU cell lines (sCLU: false discovery rate (p_FDR_) < 0.001; S100B: p_FDR_ < 0.001). At hypoxic conditions, only sCLU demonstrated significantly elevated ATP levels compared to mock cells (p_FDR_ < 0.01). Viability of nCLU and S100B cell lines was with 85% insignificantly higher than that of mock cells with 84%. When exposed to oxidative stress, ATP levels of the sCLU and S100B cell lines were significantly higher than in the mock cell line (sCLU: p_FDR_ < 0.01; S100B: p_FDR_ < 0.01), while the nCLU cell line exhibited a similar ATP concentration as did the mock cell line.

### Transcriptome sequencing of transfected cell lines

**Fig. 2:**
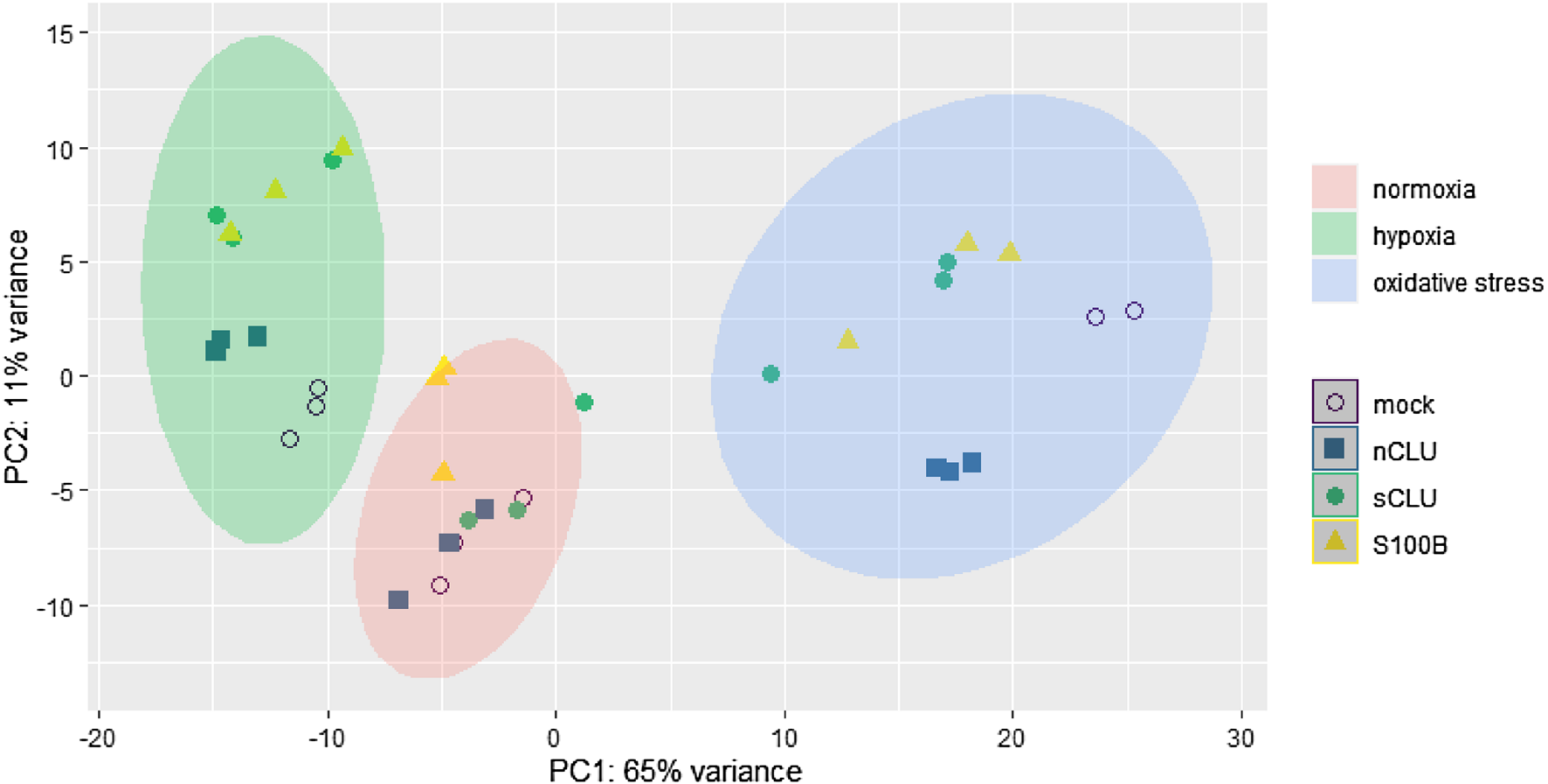
Principal component analysis of transcriptomes from transfected cell lines. Obtained reads from transfected cell lines were mapped against the mouse reference genome (GRCm39) and read counts analysed using DESeq2. Gene expressions mostly clustered according to oxygen conditions (normoxia, hypoxia, oxidative stress)

**Fig. 3:**
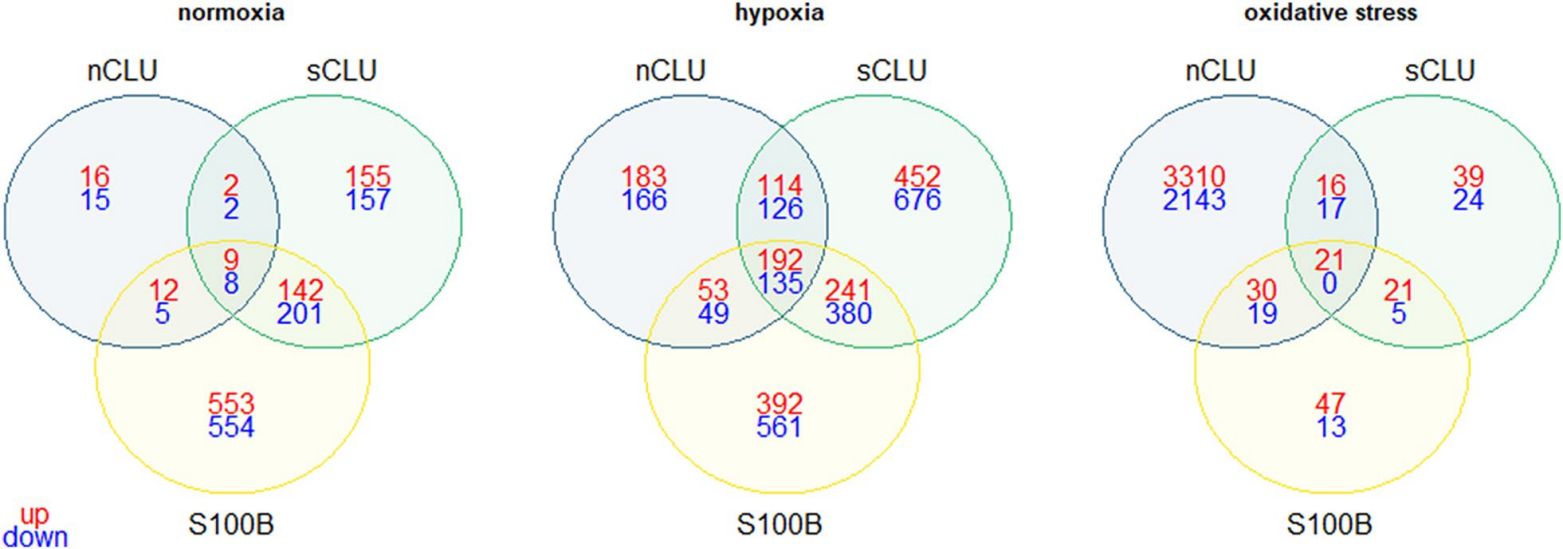
Venn diagrams of differentially expressed genes in transfected cell lines. Illustrated are the significantly upregulated [red, binary logarithm of fold change (log2FC) > 0] and downregulated (blue, log2FC < 0) genes, with transcripts per million (TPM) > 1 in transfected cell lines for each stress condition, compared to the mock cell line at the respective condition

Three replicates per cell line and condition were sequenced, with the exception of the mock cell line at oxidative stress conditions with two replicates. An average of 51 million RNA-Seq reads per sample were generated, with a minimum of 27 million and a maximum of 82 million reads per sample. Around 75% of all reads mapped to the GRCm39 mouse reference genome (Additional file 1: Table S1) across all cell lines. Cells were compared to the mock cell line at the respective stress condition, to identify differentially expressed genes (DEGs). Principal component analysis of DEGs revealed that cells clustered according to stress conditions (Fig. 2). Hypoxia and oxidative stress elicited very distinct responses that were well-distinguishable. Still, within stress conditions, differences in gene expression for transfected cell lines could be determined (Fig. 3).

At normoxia, the nCLU cell line demonstrated the least variable DEGs (p < 0.05, TPM > 1), with only 69 DEGs, while the sCLU and S100B cell lines exhibited 676 and 1484 DEGs, respectively (Fig. 3). At hypoxia, numbers were more similar across cell lines, with 1018, 2316 and 2003 DEGs for nCLU, sCLU and S100B cell lines, respectively. Proportions shifted, when at oxidative stress the nCLU cell line displayed 5556 DEGs whereas sCLU and S100B cells only had 143 and 156 DEGs. The sCLU and S100B cell lines shared a substantial amount of DEGs at normoxia (142 up- and 201 downregulated genes), while the nCLU cell line only contributed few genes to the shared DEG pool of all cell lines (9 up- and 8 downregulated genes). The overlap of DEGs between cell lines was larger at hypoxia (192 up- and 135 downregulated genes), but was almost non-existent at oxidative stress (21 upregulated genes).

### Transcriptome response of the nCLU-transfected cell line

#### The nCLU-transfected cells exhibit minor gene expression differences at normoxic conditions

**Fig. 4:**
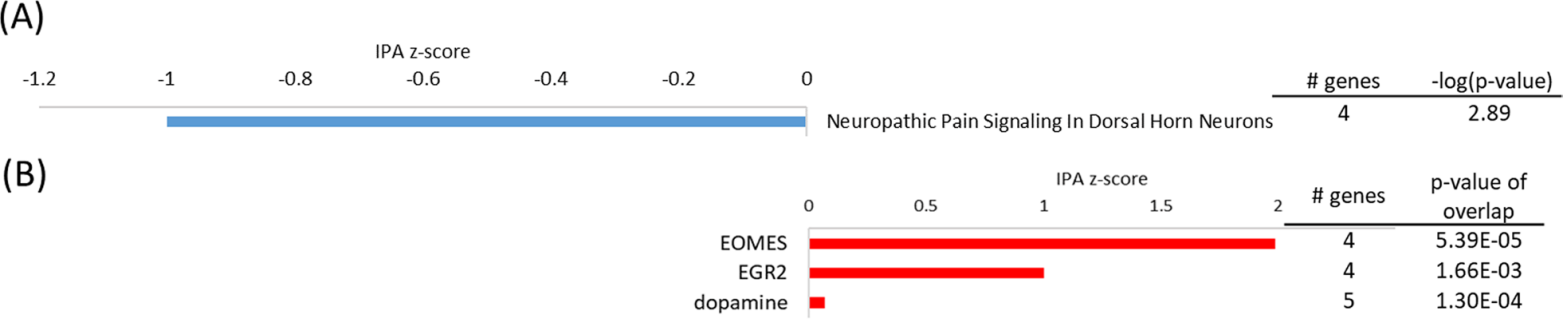
Top pathways and upstream regulators for the nCLU cell line at normoxia. **A** Top IPA canonical pathways activated (red, positive z-score) and inhibited (blue, negative z-score); **B** Top upstream regulators activated (red, positive z-score) and inhibited (blue, negative z-score) in the nCLU cell line at normoxia in comparison to the mock cell line

In normoxia, the nCLU cell line demonstrated the least differentially expressed genes (DEGs) in comparison to the mock cell line. The only enriched pathway was the inhibited n*europathic pain signaling in dorsal horn neurons* [z-score (z) = − 1, − log(p) = 2.9] (Fig. 4) with an upregulated potassium channel (KCNN3) within this pathway [binary logarithm of fold change (log2FC) = 1.19]. Some developmental genes also showed high expression, demonstrated by activation of upstream regulator eomesodermin (EOMES, z = 2.0, p < 0.0001). Homozygous silencing of EOMES leads to neurodevelopmental disorders such as microcephaly in the human brain [3] and full knockout leads to embryonic lethality in mice [90]. One of EOMES’ targets, semaphorin receptor plexin A4 (PLXNA4, (log2FC = 1.01), as well as another gene involved in axon guidance, ephrin type-A receptor 8 (EPHA8) exhibited high expression levels (log2FC = 2.16). Axon guidance may be an important process for development of the nervous system [46]. In contrast, the expression of opioid receptor delta 1 (OPRD1) was decreased in nCLU cells after normoxia (log2FC = − 1.49). In mouse astrocyte cell culture, activation of the delta opioid receptor increased expression of excitatory amino acid transporters, suggesting a role in glutamate uptake and prevention of glutamate-induced neuroexcitotoxicity [63]. Similar neuroprotection by delta opioid receptor activation has been found in mouse neuronal cell culture, which attenuated neuronal injury in normoxic and hypoxic conditions [37, 104]. Downregulation of OPRD1 may therefore indicate reduced capacity of nCLU-transfected cells to respond to stress conditions.

#### The nCLU-transfected cells demonstrate elevated hypoxia response

**Fig. 5:**
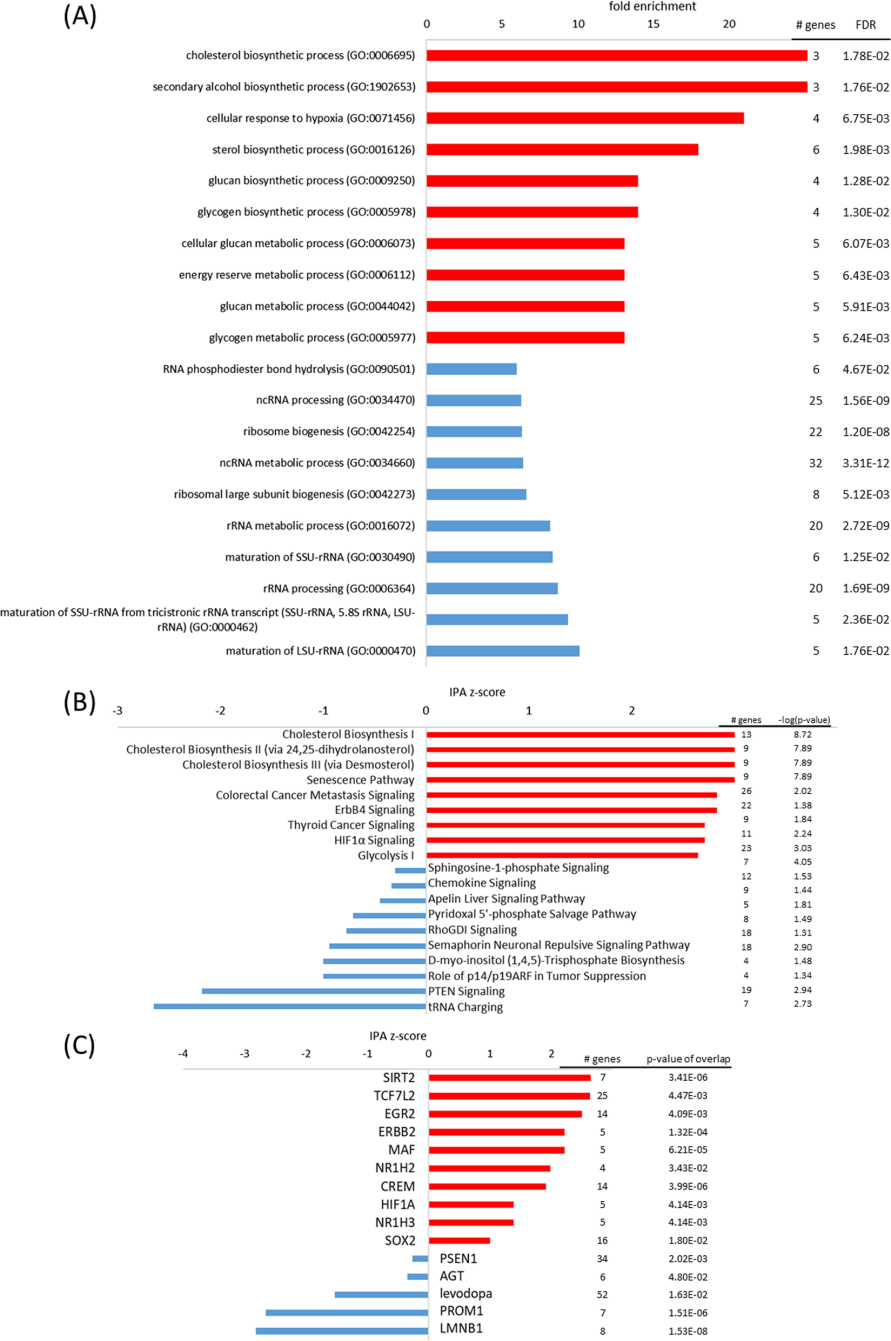
Top pathways and upstream regulators for the nCLU cell line at hypoxia. **A** Top GO terms enriched in upregulated (red) and downregulated (blue) genes; **B** top IPA canonical pathways activated (red, positive z-score) and inhibited (blue, negative z-score); **C** top upstream regulators activated (red, positive z-score) and inhibited (blue, negative z-score) in the nCLU cell line at hypoxia in comparison to the mock cell line

When exposed to hypoxia, the nCLU-transfected neuronal cells demonstrated elevated hypoxia response pathways in comparison to the mock cells (Fig. 5). Nevertheless, the mock cells displayed a hypoxia response as well. When comparing its gene expression in hypoxic conditions to normoxic conditions, *cellular response to hypoxia* was among top 10 enriched pathways (FE = 15.89, pFDR < 0.05). Even when only looking at highly differentially expressed genes (log2fc > 1 or log2fc < − 1) *cellular response to hypoxia* was among top 10 enriched pathways (FE > 100, pFDR < 0.05). However, hypoxia response of the nCLU-transfected cells appeared to be even stronger in comparison to mock cells.

In hypoxia, most eukaryotic cells can shift their primary metabolic strategy from predominantly mitochondrial respiration towards increased glycolysis [51]. However, it is not clear if neurons are able to undergo such a shift in metabolism [15]. GO term *glycolytic process* [fold enrichment (FE) = 9.8, false discovery rate (p_FDR_) < 0.01], as well as IPA pathways *glycolysis* I (z = 2.6, − log(p) = 4.1) and *gluconeogenesis* (z = 1.6, − log(p) = 3.1) were enhanced in nCLU-transfected cells. Additionally, GO term *glycogen biosynthetic process* (FE = 14.0, p_FDR_ < 0.05) and IPA pathway *glycogen biosynthesis* (− log(p) = 3.0) were increased in the nCLU-transfected cells, which is a common response to hypoxia [80]. Thus, enzymes for glycogen metabolism which mainly promote glycogen accumulation (e.g., glucan phosphatase EPM2A, log2FC = 1.03) [86] were upregulated in the nCLU-transfected cell lines. Glycogen has been demonstrated to protect cerebellar and cortical mouse neurons from hypoxic stress-induced cell death in cell culture [91] and glycogen storage is increased in the seal brain [14, 50], which illustrates its significance in dealing with hypoxia. In a transcriptome analysis, glycogenolysis-associated genes were, thus, found to be upregulated in hooded seal neurons compared to neurons of mice [30]. In general, energy metabolism through glycogen biosynthesis and glycolysis were enhanced in the nCLU-transfected cells in hypoxic conditions.

Response to hypoxia is substantially mediated by the hypoxia-inducible factor (HIF1), which is a master regulator that activates the transcription of many genes involved in energy metabolism, apoptosis and oxygen delivery. In the present study, nCLU transfected cell lines increased *HIF1A signaling* (z = 2.7, − log(p) = 3.0), while HIF1A was also found to be an upstream regulator (z = 1.4, p < 0.01). Additionally, in GO analyses, *cellular response to hypoxia* was enhanced in the nCLU cell line (FE = 21.0, p_FDR_ < 0.01). However, as in normoxic conditions, OPRD1 demonstrated low expression in the nCLU cell line (log2FC = − 1.32), which might be disadvantageous in dealing with hypoxic conditions.

#### The oxidative stress response of the nCLU cell line is characterized by mitochondrial dysregulation

**Fig. 6:**
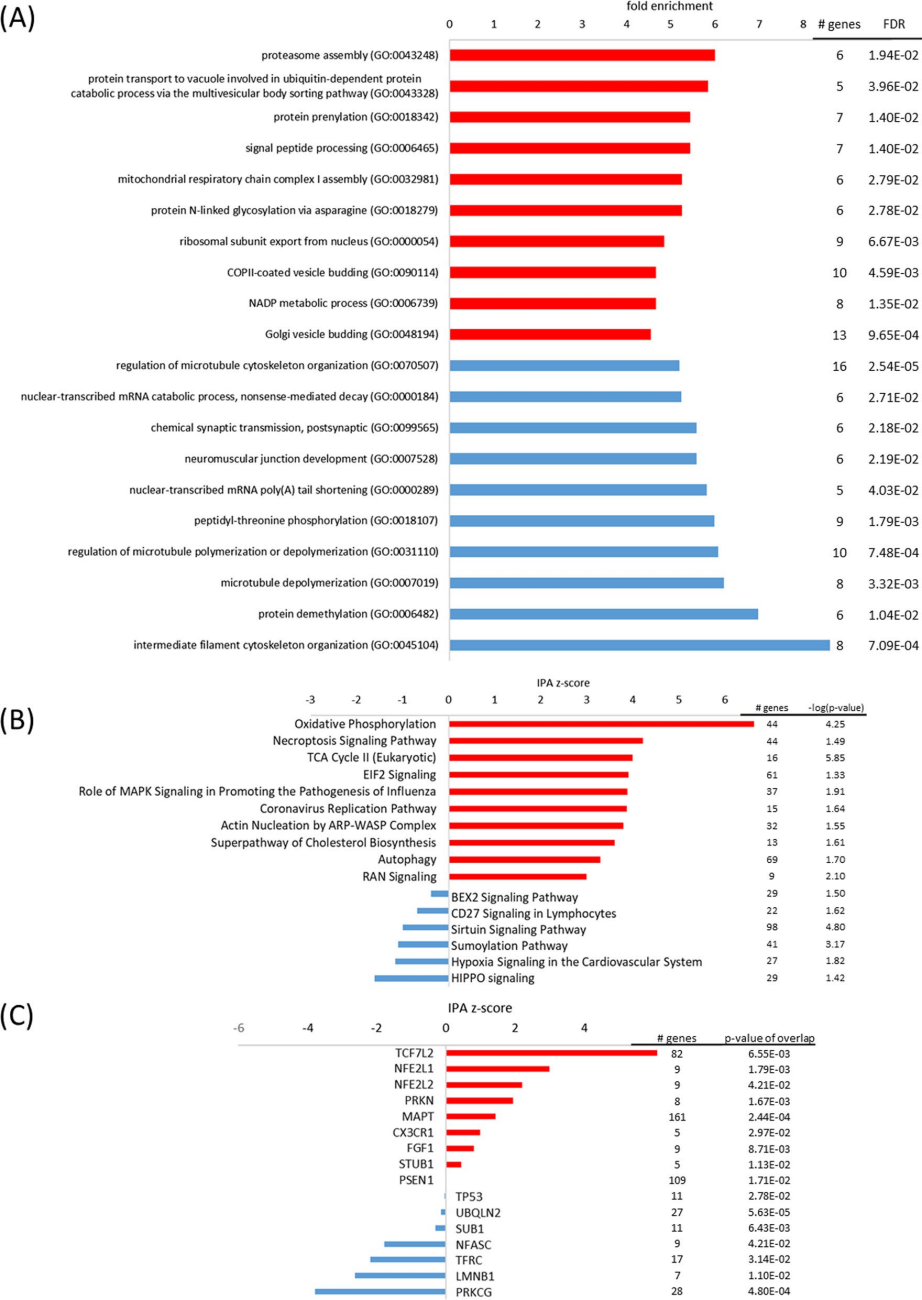
Top pathways and upstream regulators for the nCLU cell line at oxidative stress. **A** Top GO terms enriched in upregulated (red) and downregulated (blue) genes; **B** top IPA canonical pathways activated (red, positive z-score) and inhibited (blue, negative z-score); **C** top upstream regulators activated (red, positive z-score) and inhibited (blue, negative z-score) in the nCLU cell line at oxidative stress in comparison to the mock cell line

Oxidative stress is an inevitable by-product of oxidative metabolism and reflects a state of imbalance between reactive oxygen species (ROS) and substances that are involved in their detoxification, which may cause damage to proteins, lipids and DNA. Diving mammals may counteract oxidative stress through elevated antioxidant levels in its brain [20, 30]. At oxidative stress conditions, antioxidants, such as the glutathione S-transferase alpha 4 (GSTA4, log2FC = 1.80), thioredoxin 1 (TXN1, log2FC = 1.40), peroxiredoxin 3 (PRDX3, log2FC = 1.40), selenoprotein F (SELENOF, log2FC = 1.27) and the putative glutathione peroxidase 8 (GPX8, log2FC = 1.00) were increased in the nCLU cell line, which may partly be ascribed to activation of nuclear factor, erythroid 2-like 2 (NFE2l2, also abbreviated as NRF2, z = 2.2, p < 0.05) as upstream regulator. The transcription factor NRF2 plays a pivotal role controlling the expression of antioxidant genes that exert neuroprotective functions [92]. Nevertheless, the nCLU cell line exhibited a profound *response to oxidative stress* (FE = 2.8, p_FDR_ < 0.05), characterized by *mitochondrial dysfunction* (− log(p) = 8.1), enhanced *oxidative phosphorylation* (z = 6.6, − log(p) = 4.3) and *TCA cycle* (z = 4.0, − log(p) = 5.9) (Fig. 6). Mitochondrial complexes such as cytochrome c oxidase subunits COX7A2 (log2FC = 1.91), COX4I1 (log2FC = 1.28), COX5B (log2FC = 1.10), COX7C (log2FC = 1.09), NADH:ubiquinone oxidoreductase subunits NDUFB1 (log2FC = 1.37), NDUFC2 (log2FC = 1.36), NDUFB9 (log2FC = 1.34), NDUFV2 (log2FC = 1.20), NDUFA7 (log2FC = 1.12), NDUFS4 (log2FC = 1.06), NDUFA9 (log2FC = 1.03), isocitrate dehydrogenases IDH3A (log2FC = 1.08), IDH1 (log2FC = 1.05) and succinate dehydrogenase subunit SDHB (log2FC = 1.09) exhibited high expression levels. Dysregulated aerobic respiration may have led to *apoptotic mitochondrial changes* (FE = 4.3, p_FDR_ < 0.05), increased *proteasome assembly* (FE = 6.0, p_FDR_ < 0.05) and *autophagy* (z = 3.3, − log(p) = 1.7), which might serve to clear cells from dysfunctional mitochondria. Important genes involved in autophagy processes such as BCL2 interacting protein 3 (BNIP3, log2FC = 1.11), VPS35 retromer complex component (VPS35, log2FC = 1.01), as well as FUN14 domain containing 1 (FUNDC1, log2FC = 1.76) were upregulated in the oxidative-stress-challenged nCLU cell line. Ultimately, autophagy processes and redox imbalance may have induced *ferroptosis* (z = 1.2, − log(p) = 1.9), which is an iron-dependent form of non-apoptotic cell death [97] and *necroptosis signaling pathway* (z = 4.2, − log(p) = 1.5), another form of non-apoptotic cell death [74]. In accordance, low expression of glial cell line derived neurotrophic factor (GDNF, log2FC = − 1.45) may reduce survival pathways [12]. In summary, the nCLU-transfected cells attempted to counter the deleterious effects of dysregulated mitochondrial aerobic respiration by increasing antioxidant expression, but ultimately experienced elevated cell death at oxidative stress conditions.

### Transcriptome response of the sCLU-transfected cell line

#### The sCLU-transfected cells promote glycolytic capacity and protein folding at normoxia

In contrast to the nCLU cell line, the sCLU-transfected cells already demonstrated substantial differences in gene expression at normoxic conditions, which included energy metabolism and autophagy processes. Adenosine triphosphate (ATP) provides the energy necessary to drive all energy-demanding processes in living cells. Elevated ATP levels of sCLU-transfected cells have already been demonstrated in cell viability assays [31] and were confirmed in this study. At aerobic conditions, the main ATP production usually takes place at the mitochondrial electron transport chain. However, this process is also associated with the generation of ROS, which can be detrimental in high concentrations. The high expression of pyruvate dehydrogenase kinase 1 (PDK1, log2FC = 1.20) in sCLU cells might inactivate pyruvate dehydrogenase and consequently inhibit the first step of the citric acid cycle. In carcinoma and fibroblast cell cultures, overexpression of PDK1 shifted ATP production from mitochondrial respiration to glycolysis, thereby attenuating hypoxic ROS generation and rescuing cells from hypoxia-induced apoptosis [52, 79]. Additionally, low expression of myeloid translocation gene 16 product (MTG16, also abbreviated as CBFA2T3, log2FC = − 1.14) was observed in the sCLU cell line. MTG16 reduced the expression of PDK1 and genes involved in glycolysis in lymphoma cells [57], and reduced levels of MTG16 might therefore indicate enhanced glycolytic capacity. Consistent with this, sCLU expression correlated with high expression of the monocarboxylate transporter 4 (MCT4, also abbreviated as SLC16A3, log2FC = 1.20). MCT4 has an important role in tissues reliant on glycolysis [36], by facilitating lactate efflux and preventing pyruvate efflux, thereby enabling conversion of pyruvate to lactate and regeneration of NADH for glycolysis [36]. MCT4 was also upregulated in hooded seal brain slices that were exposed to hypoxia and reoxygenation in vitro [39] and MCT4 was more highly expressed in hooded seal than in mouse neurons [30]. Exported lactate by MCT4 might be further metabolized by neighboring astrocytes in the hooded seal brain that exhibit high levels of lactate dehydrogenase b (LDHB) [40], as suggested by the ‘reverse lactate shuttle’ hypothesis, first presented by Mitz et al. [73]. Additionally, aerobic metabolism was found to be decreased in the visual cortices of hooded seals compared to ferrets [20]. In contrast, Geßner et al. [30] concluded that mitochondrial function and numbers may have been enhanced, while glycolytic capacity was slightly lower, in neurons of the hooded seal compared to mice. Arguably, these differences might be related to the choice of the non-diving model organism, i.e., ferrets vs mice, which are known to maintain quite different basal metabolic rates [30]. Further, we here consider the effect of particular genes, whereas Geßner et al. [30] analysed the neuronal transcriptome as a whole. In summary, alterations of pathways by sCLU during normoxia may indicate a preparation or pre-adaptation of hooded seals to upcoming diving-associated stress conditions, such as low oxygen levels or ROS production. To prepare for these conditions, capacity for aerobic respiration might be decreased and capacity for anaerobic glycolysis might be increased.

**Fig. 7:**
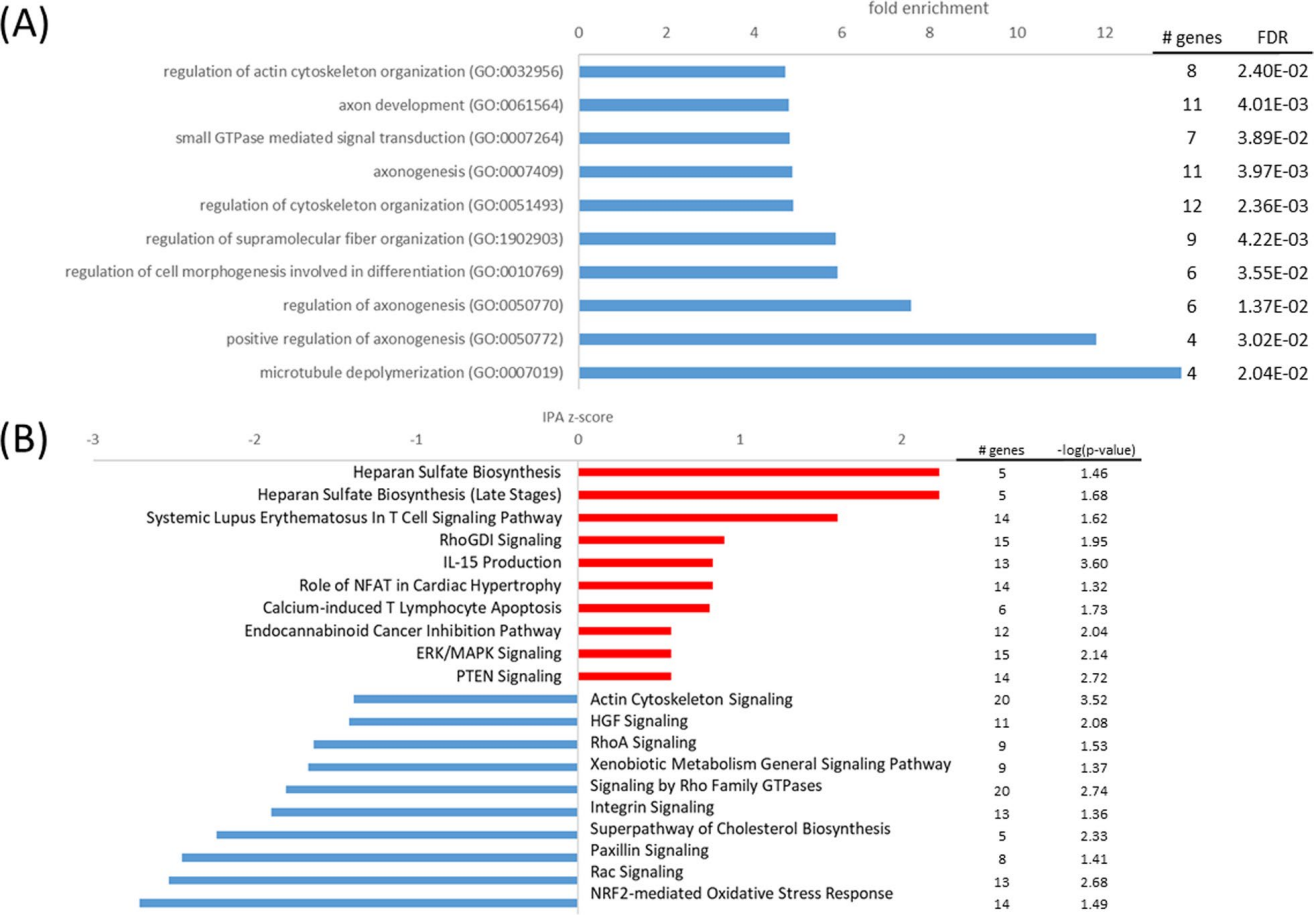
Top pathways and upstream regulators for the sCLU cell line at normoxia. **A** Top GO terms enriched in upregulated (red) and downregulated (blue) genes; **B** top IPA canonical pathways activated (red, positive z-score) and inhibited (blue, negative z-score) in the sCLU cell line at normoxia in comparison to the mock cell line

Cellular stress responses comprise mechanisms that minimize acute damage and promote cell survival. Oxidative stress might denature proteins, thereby disrupting protein homeostasis (proteostasis) necessary for biological function and cell metabolism [10]. Chaperones can help defend the cell against damage by facilitating protein folding, ensuring that proteins assume their necessary shape. Misfolded proteins may be degraded by proteasomes or autophagy, to remove potentially toxic aggregates. Clusterin (CLU) may play an important role as stress-induced secreted chaperone protein, mediating proteasomal degradation of misfolded proteins [45, 93], and CLU is known to protect neuronal cells against intracellular protein aggregation and cytotoxicity [34]. Interestingly, in IPA analyses the *endoplasmic reticulum stress pathway* (z = 0.4, − log(p) = 5.23) and *unfolded protein response* (z = 0.3, − log(p) = 2.84) were slightly activated in the normoxic sCLU-transfected cells, suggesting the contribution of CLU to protein homeostasis in the hooded seal brain. In addition, the chaperone peptidylprolyl isomerase C (PPIC, log2FC = 1.15) was upregulated in sCLU cells, which may also be important for coping with oxidative stress [58, 67]. Additionally, a*utophagy* (z = 0.2, − log(p) = 2.51), which promotes degradation of damaged proteins was slightly activated in the sCLU-transfected cell line. In particular, BNIP3, which is necessary for clearing dysfunctional mitochondria with low membrane potential and reducing the buildup of ROS to promote cell survival [78], was observed to be more highly expressed in the sCLU cell line (log2FC = 1.33). Furthermore, *NRF2-mediated oxidative stress response* (z = − 2.7, − log(p) = 1.5) (Fig. 7), which coordinates the basal and stress-inducible activation of a vast array of cytoprotective genes, like antioxidants [92], was downregulated in sCLU cells. A reduction of this pathway may imply decreased stress and reduced necessity to detoxify ROS. In accordance with our results, Geßner et al. [31] found no increased caspase activity and ROS amount for sCLU-transfected HN33 cells at normoxia, indicating no increased stress. In conclusion, sCLU might contribute to the stress tolerance of the hooded seal brain by improving autophagy and protein folding pathways as well as glycolytic capacity.

#### The sCLU cell line exhibits elevated hypoxia response

**Fig. 8:**
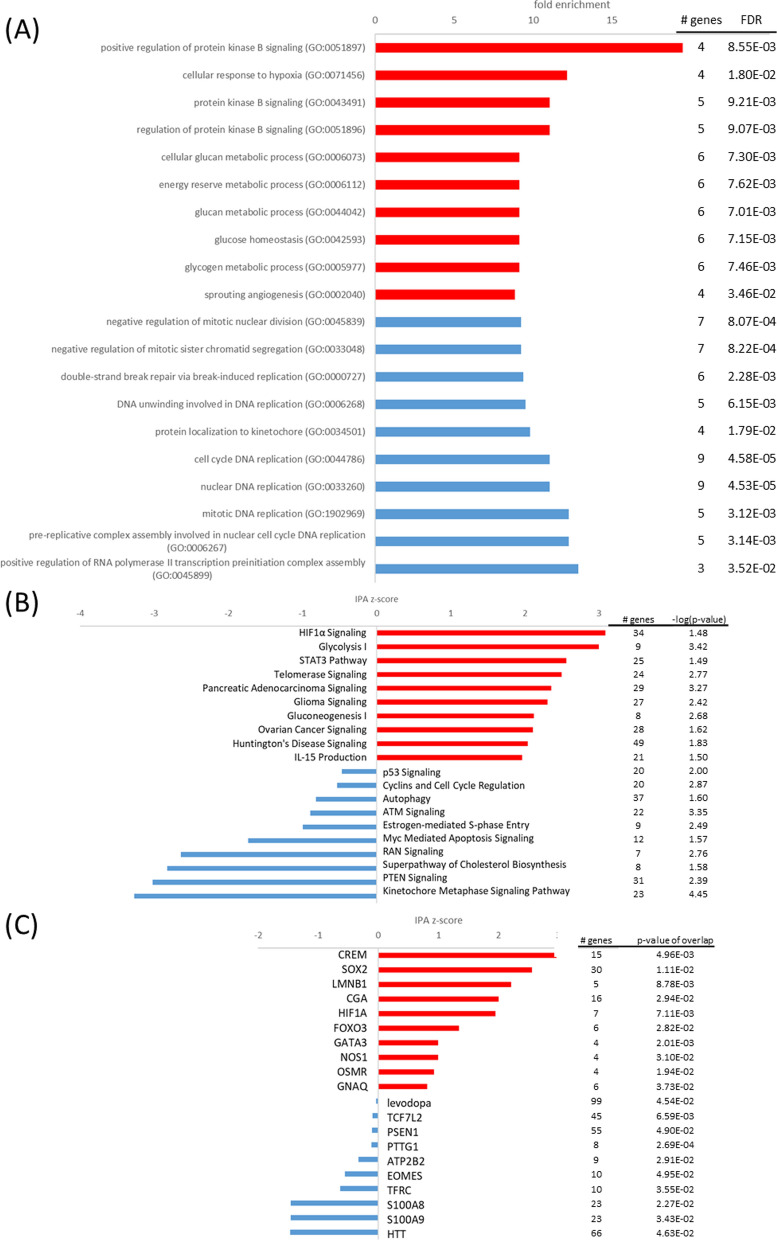
Top pathways and upstream regulators for the sCLU cell line at hypoxia. **A** Top GO terms enriched in upregulated (red) and downregulated (blue) genes; **B** top IPA canonical pathways activated (red, positive z-score) and inhibited (blue, negative z-score); **C** top upstream regulators activated (red, positive z-score) and inhibited (blue, negative z-score) in the sCLU cell line at hypoxia in comparison to the mock cell line

When exposed to hypoxic stress, the sCLU cell line exhibited a similar transcriptome response to the nCLU-transfected cells. IPA pathways *glycolysis* I (z = 3.0, − log(p) = 3.4), *gluconeogenesis* (z = 2.1, − log(p) = 2.7) and *glycogen biosynthesis* (− log(p) = 2.0) and GO terms *glycolytic process* (FE = 5.7, p_FDR_ < 0.05) and *glycogen biosynthetic process* (FE = 8.1, p_FDR_ < 0.05) were enhanced (Fig. 8). The related glycogen storing enzyme EPM2A, important for accurate accumulation of glycogen [86], also demonstrated increased transcription (log2FC = 2.07). On the other hand, *aerobic respiration* (FE = 3.3, p_FDR_ < 0.05) was decreased in sCLU-transfected cells. Aerobic respiration may have been reduced by high expression of PDK1 (log2FC = 1.01) and low expression of MTG16 (log2FC = − 0.46) in sCLU-transfected cells, which inhibit the first step of the TCA cycle and improve glycolytic capacity [52, 57, 79]. However, regulation of PDK1 and MTG16 genes was not as strong as at normoxia. This was probably related to that mock cells to some extent also downregulated aerobic respiration at hypoxia and the difference between these cell lines may have become less distinct.

GO term *cellular response to hypoxia* (FE = 21.0, p_FDR_ < 0.05) in the sCLU cell line may have also been mediated by HIF1A as upstream regulator (z = 2.0, p < 0.01) and *HIF1A signaling* (z = 3.1, − log(p) = 1.5). Furthermore, egl-9 family hypoxia-inducible factor 1 (EGLN1) was highly expressed (log2FC = 1.06), which hydroxylates HIF proteins and thereby targets them for degradation [26]. However, the hydroxylation reaction might have been attenuated by limited oxygen availability. In addition to high expression of BNIP3 (log2FC = 1.36), HIF1-upregulated mitochondria-localized glutamic acid rich protein (MGARP, log2FC = 1.33) was highly expressed in sCLU cells and might further support mitophagy [43], which may reduce deleterious ROS by clearance of dysfunctional mitochondria. Additionally, upregulation of PPIC (log2FC = 2.06) may have further improved protein folding and reduced ER stress in the sCLU-transfected cell line [58, 67]. According to described neurotrophic functions, *intrinsic apoptotic signaling* (FE = 3.6, p_FDR_ < 0.05) as well as *MYC mediated apoptosis* (z = − 1.7, − log(p) = 1.6) were found to be downregulated in the sCLU cell line. Decreased apoptosis is in line with previous findings, e.g. as reflected by decreased caspase activity and phosphatidylserine exposure in transfected cells [31]. Overall, sCLU cells might enhance stress resistance and reduce apoptosis in response to hypoxia.

#### The sCLU-transfected cells show limited transcriptome response at oxidative stress despite elevated viability

When applying oxidative stress, the sCLU cell line exhibited a limited DEG response. This seems counterintuitive, since sCLU cells had a significantly higher viability in oxidative stress than nCLU and mock cells [31]. Normoxic (i.e., constitutional) differences in gene expression might therefore already have prepared sCLU-transfected cells for oxidative stress, indicating a pre-adaptive response to upcoming stress conditions.

**Fig. 9:**
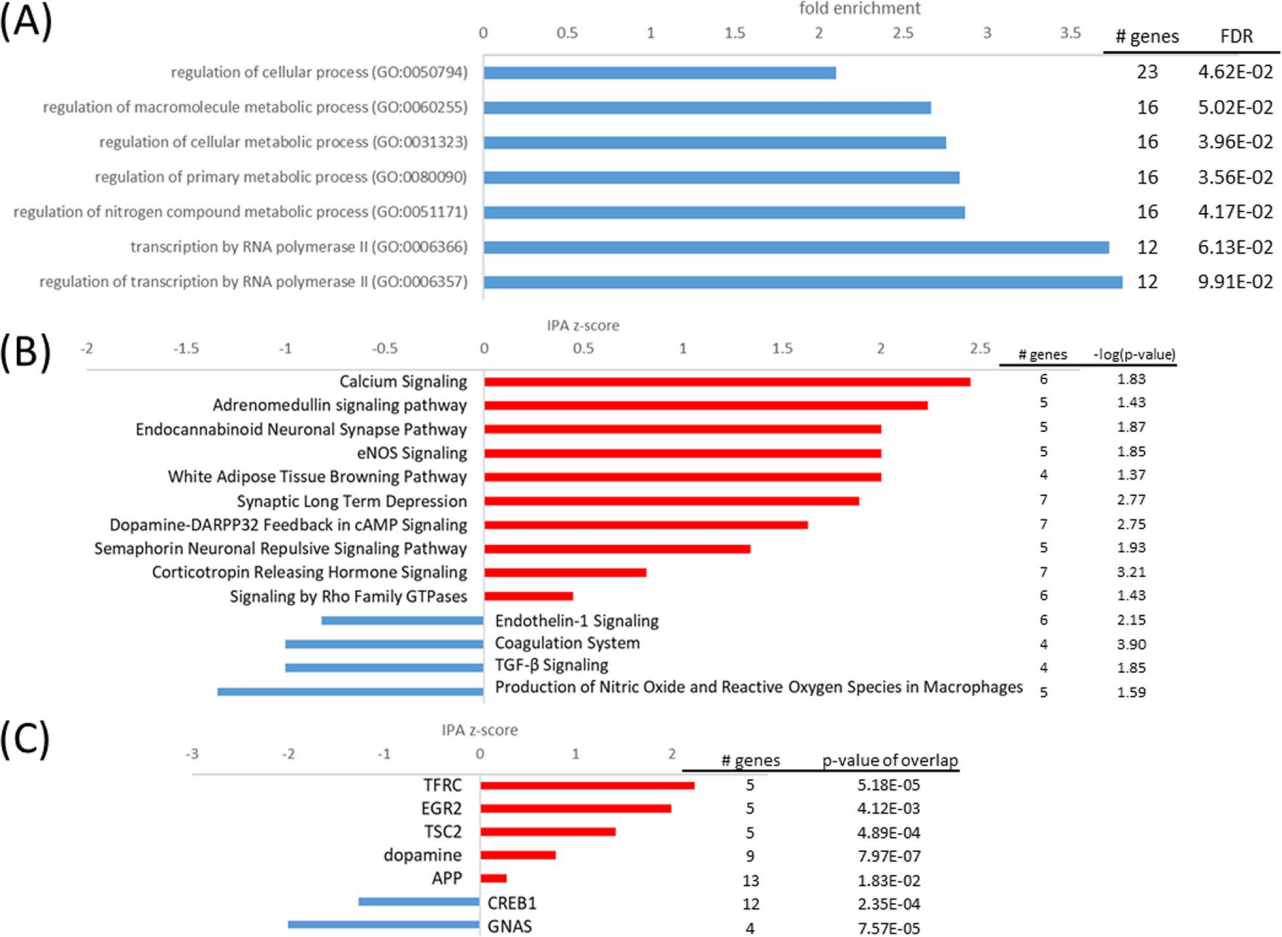
Top pathways and upstream regulators for the sCLU cell line at oxidative stress. **A** Top GO terms enriched in upregulated (red) and downregulated (blue) genes; **B** top IPA canonical pathways activated (red, positive z-score) and inhibited (blue, negative z-score); **C** top upstream regulators activated (red, positive z-score) and inhibited (blue, negative z-score) in the sCLU cell line at oxidative stress in comparison to the mock cell line

Nevertheless, the upregulation of some genes might assist sCLU in protein folding such as cAMP responsive element binding protein 3-like 2 (CREB3L2, log2FC = 1.26) and PPIC (log2FC = 2.17). CREB3L2 protected cells from ER stress-induced death in a neuroblastoma cell line [55], while the chaperone PPIC might have further promoted protein folding and reduce oxidative stress [67]. Additionally, pathways involved in synaptic signaling were elevated in the sCLU cell line at oxidative stress such as *calcium signaling* (z = 2.5, − log(p) = 1.8), *semaphorin neuronal repulsive signaling* (z = 1.3, − log(p) = 1.9) and *synaptic long term depression* (z = 1.9, − log(p) = 2.8) (Fig. 9). High expression of voltage-dependent calcium channels CACNA1G (log2FC = 1.48) and CACNA1I (log2FC = 1.32) may have facilitated calcium flux and subsequent binding of calcium to synaptotagmin I (SYT1, log2FC = 1.33), which may have triggered neurotransmitter release at the synapse [22]. Especially serotonin may have functioned as neurotransmitter in the oxidative stress-exposed sCLU cell line. Tryptophan hydroxylase 2 (TPH2, log2FC = 1.74), which catalyzes the first rate-limiting step in serotonin biosynthesis [41], as well as the serotonin receptor HTR3A (log2FC = 1.43) demonstrated increased expression. Binding of serotonin to HTR3A causes fast, depolarizing responses in neurons [5], but may also regulate the development of the mammalian central nervous system [19]. Described genes might support observed decrease in lipid peroxidation and caspase activity in a previous study [31] and protect sCLU-transfected cells from oxidative stress induced cell death. The majority of preventive measures in sCLU cells though may have already been taken at normoxic conditions.

### Transcriptome response of the S100B-transfected cell line

#### The S100B-transfected cells reduce synaptic signaling pathways at normoxia

Similar to the sCLU cell line, the S100B-transfected cells exhibited elevated ATP levels at normoxic conditions, which is in accordance to a previous study [31]. Likewise, *glycolytic process* (FE = 6.6, p_FDR_ < 0.05) and MCT4 expression (log2FC = 1.87) were elevated in the S100B cells at normoxia, while the TCA cycle may have been inhibited by high expression of PDK1 (log2FC = 1.14). Consequently, capacity for glycolytic metabolism may have been increased and aerobic respiration decreased in the S100B cell line at normoxic conditions.

Furthermore, S100B may play a role in neurodegeneration or neuroprotection [27]. Although genes associated with the GO term *neuron development* (FE = 2.3, p_FDR_ < 0.05) demonstrated reduced expression in S100B-transfected cells, increased expression of neurotrophic factors such as growth-associated protein 43 (GAP43, log2FC = 1.52) and brain-derived neurotrophic factor (BDNF, log2FC = 0.83) might enhance neuron growth and survival. GAP43 may regulate synaptic plasticity and neurite outgrowth [95] and is also an important mediator of the neuroprotective effects of BDNF in connection with excitotoxicity [35]. The growth factor BDNF is one of the most widely distributed and extensively studied neurotrophins in the mammalian brain [56], and is associated with neuronal maintenance, survival, plasticity, and neurotransmitter regulation [32]. Furthermore, the neurotrophic growth factor pleiotrophin (PTN, log2FC = 0.37) and its receptor anaplastic lymphoma kinase (ALK, log2FC = 1.20) were upregulated in S100B-transfected cells [48]. Loss of PTN in pericyte-ablated mice has been linked to a rapid neurodegeneration cascade [75], which illustrates its role in neuroprotection. Additionally, tissue-type plasminogen activator (PLAT, log2FC = 1.03) and a PLAT inhibitor (SERPINI1, log2FC = 1.07), which are involved in synaptic plasticity [9] and have been reported to facilitate neuron survival depending on concentration and isoform [11], were upregulated in S100B cells. In summary, although most genes involved in neuron development were slightly downregulated in normoxic S100B cells, highly upregulated important neurotrophic factors may have promoted neuronal plasticity and survival.

**Fig. 10:**
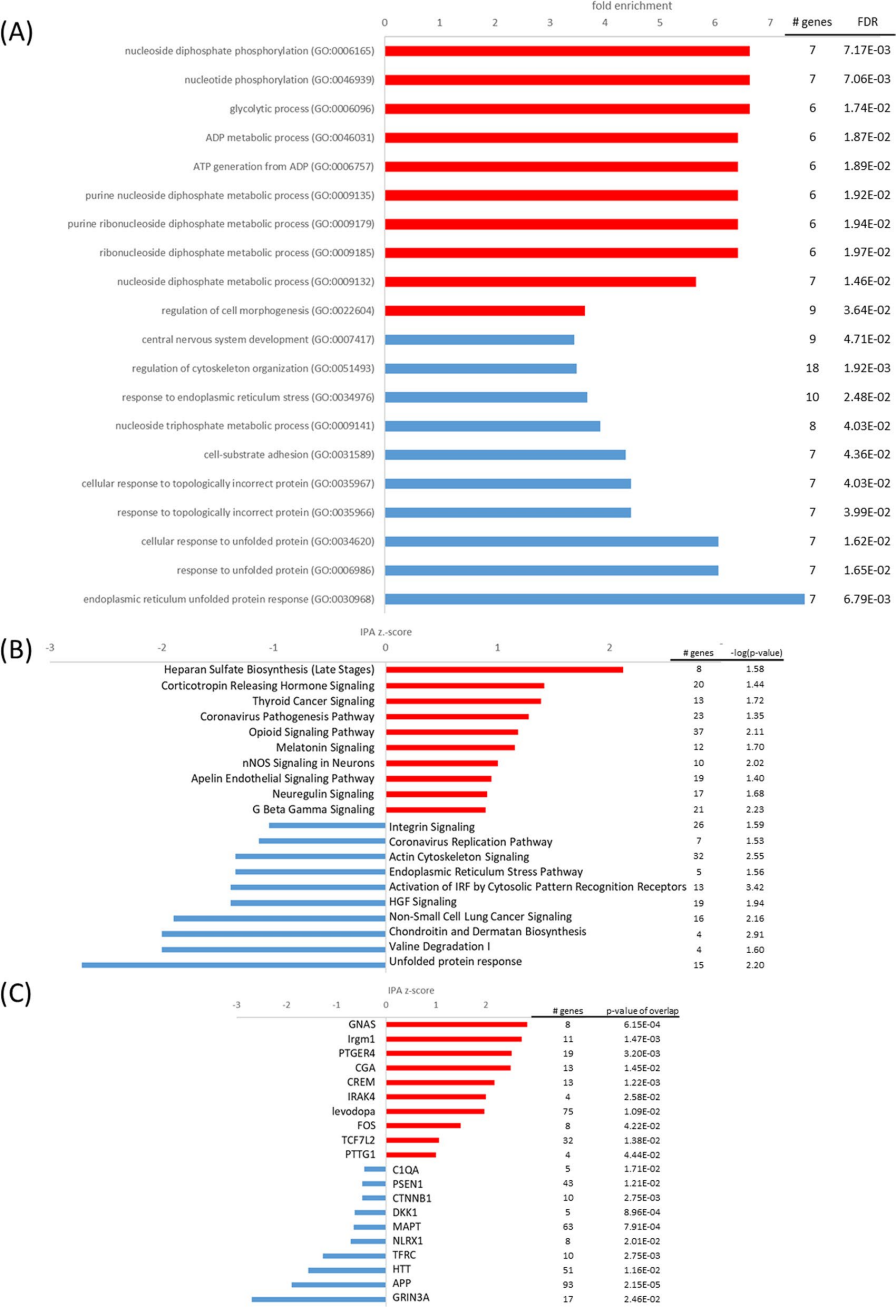
Top pathways and upstream regulators for the S100B cell line at normoxia. **A** Top GO terms enriched in upregulated (red) and downregulated (blue) genes; **B** top IPA canonical pathways activated (red, positive z-score) and inhibited (blue, negative z-score); **C** top upstream regulators activated (red, positive z-score) and inhibited (blue, negative z-score) in the S100B cell line at normoxia in comparison to the mock cell line

During synaptic signaling, the presynaptic neuron membrane at the synapse depolarizes, causing influx of calcium ions and release of neurotransmitters. Reestablishing the resting membrane potential, calcium gradient and neurotransmitter levels, are all highly energy-intensive processes in the brain. S100B is involved in calcium homeostasis and thereby presumably also in regulating synaptic plasticity [16]. It has been suggested that its high expression in marine mammals may help prevent excitotoxicity by reducing free intracellular Ca^2+^ and thereby attenuate continued release of glutamate and other neurotransmitters [28]. In the S100B-transfected cells neurotransmitter release might be reduced by activation of *opioid signaling* (z = 1.2, − log(p) = 2.1) (Fig. 10), which can prevent calcium ion influx and facilitate potassium ion efflux, thereby causing membrane hyperpolarization and a reduced neurotransmitter release [33]. The upregulated OPRD1 (log2FC = 0.80) may have facilitated clearance of neurotransmitters from the synaptic cleft and prevent neuroexcitotoxicity [37]. Furthermore, levels of purkinje cell protein 4 (PCP4) were decreased in S100B cells (log2FC = − 1.96). PCP4 is a small calmodulin-binding protein that promotes calcium exchange and neurotransmitter release [87]. Its downregulation might further support a reduction of neurotransmitter levels in S100B cells. A previous study that compared the transcriptomes of hooded seal and mouse neurons also indicated reduced glutamatergic transmission in the seal, by reduced expression of glutamate receptors, while glutamate uptake was increased [30]. Noh et al. [77] stated that glutaminergic synapse function might have been commonly positively selected in pinnipeds, indicating its importance for the adaptation to the marine environment. In summary, S100B may contribute to the neuronal hypoxia tolerance by reducing neurotransmission and thus, our findings support the observations of Geiseler et al. [28] and Geßner et al. [30]. A reduction in neurotransmission might ultimately serve to reduce energy consumption and thereby the oxygen needs of neuronal cells.

#### The S100B cell line exhibits elevated hypoxia response

**Fig. 11:**
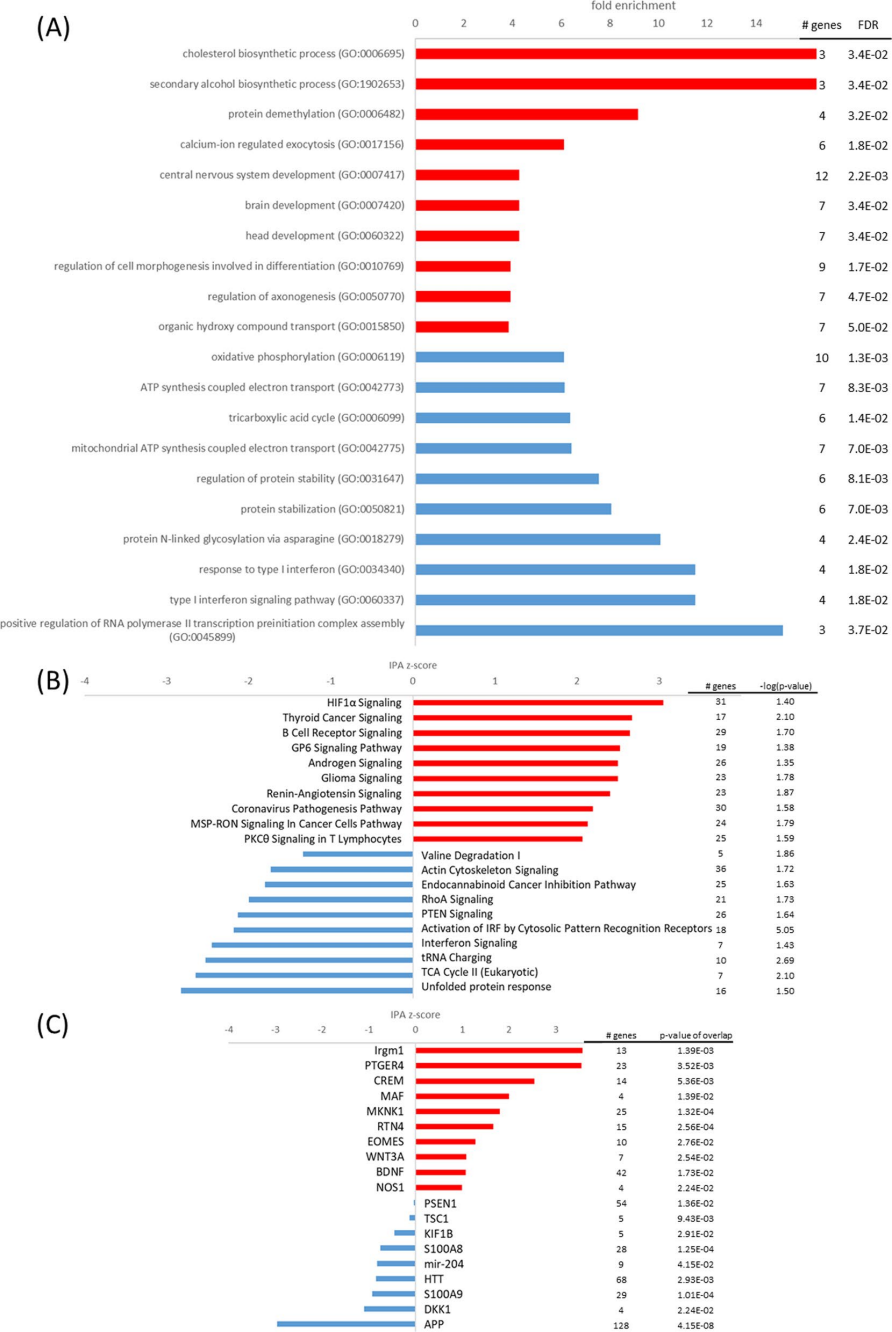
Top pathways and upstream regulators for the S100B cell line at hypoxia. **A** Top GO terms enriched in upregulated (red) and downregulated (blue) genes; **B** top IPA canonical pathways activated (red, positive z-score) and inhibited (blue, negative z-score); **C** top upstream regulators activated (red, positive z-score) and inhibited (blue, negative z-score) in the S100B cell line at hypoxia in comparison to the mock cell line

In analogy to the other transfected cell lines, hypoxia response of the S100B cell line might have been mediated by activated *HIF1A signaling* (z = 3.1, − log(p) = 1.4) (Fig. 11). GO terms *tricarboxylic acid cycle* (FE = 6.37, p_FDR_ < 0.05), *oxidative phosphorylation* (FE = 6.11, p_FDR_ < 0.01) and *aerobic respiration* (FE = 5.4, p_FDR_ < 0.01) as well as IPA pathway *TCA cycle* (z = − 2.6, − log(p) = 2.1) were decreased in S100B-transfected cells, but PDK1 upregulation (log2FC = 0.65) was not as prominent in inhibiting the first step of TCA cycle as in normoxic conditions. In accordance with the putative function of S100B GO term *calcium-ion regulated exocytosis* (FE = 6.1, p_FDR_ < 0.05) was enriched in upregulated genes of the S100B-transfected cells. Furthermore, *opioid signaling* (z = 1.9, − log(p) = 1.9) and *synaptogenesis signaling pathway* (z = 1.6, − log(p) = 1.4) were enhanced in the S100B-transfected cells when subjected to hypoxia. As mentioned before, high expression of OPRD1 (log2FC = 0.99) and low expression of PCP4 (log2FC = − 1.89) may reduce neurotransmitter levels and protect cells from hypoxia-induced excitotoxicity [37, 87]. Additionally, GAP43 (log2FC = 1.45) and BDNF (log2FC = 1.17) may facilitate neuron survival [32, 35]. According to described neurotrophic functions, *intrinsic apoptotic signaling* (FE = 4.8, p_FDR_ < 0.01) was enriched in downregulated genes of S100B-transfected cells at hypoxic conditions. Therefore, in addition to decreasing aerobic respiration, S100B may facilitate neuroprotection of neuronal cells by downregulating synaptic signaling and upregulating neurotrophic factors at hypoxic conditions.

#### The S100B cell line demonstrates limited transcriptome response at oxidative stress despite elevated viability

**Fig. 12:**
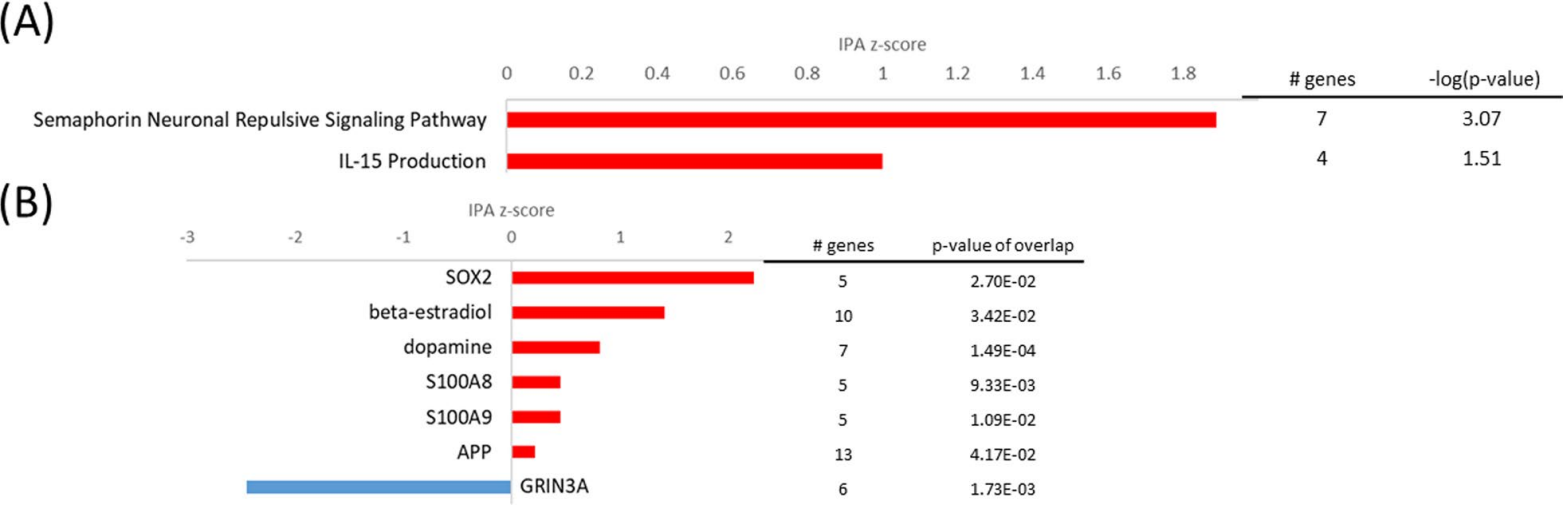
Top pathways and upstream regulators for the S100B cell line at oxidative stress. **A** Top IPA canonical pathways activated (red, positive z-score) and inhibited (blue, negative z-score); **B** top upstream regulators activated (red, positive z-score) and inhibited (blue, negative z-score) in the S100B cell line at oxidative stress in comparison to the mock cell line

Similar to the sCLU-transfected cells, the S100B cell line did not exhibit a diverse DEG response at oxidative stress. However, elevated viability [31] may point to pre-adaptive mechanisms already carried out at normoxia. When exposed to oxidative stress, the only two activated pathways in IPA analysis were *semaphorin neuronal repulsive signaling pathway* (z = 1.9, − log(p) = 3.07) and *IL-15 production* (z = 1, − log(p) = 1.51) (Fig. 12). The former already demonstrated activation at normoxia (z = 0.8, − log(p) = 2.98). Semaphorin such as SEMA6D (log2FC = 1.35) and the semaphorin co-receptor neuropilin 1 (NRP1, log2FC = 1.02) may have played an essential role in *axonal guidance signaling* (− log(p) = 2.4) and thereby overall nervous system development at oxidative stress conditions [46]. Additionally, high expression of OPRD1 (log2FC = 1.06) and low expression of PCP4 (log2FC = − 1.86) may have facilitated neuroprotection by reduction of neurotransmitter levels as mentioned before [37, 87]. These mechanisms may aid in protecting S100B-transfected cells from oxidative stress induced cell death. However, metabolic alterations at normoxia may have already prepared cells for imminent stress conditions.

## Conclusion

Clusterin (CLU) and S100B are highly expressed in the hooded seal brain and probably represent two of numerous factors that contribute to its intrinsic hypoxia tolerance. In order to investigate their potential roles, we transfected HN33 cells with soluble clusterin (sCLU), nucleus clusterin (nCLU) and S100B, subjected these cell lines to three challenges; normoxia, hypoxia and oxidative stress, and studied viability and differential gene expression (DEG) responses (Tables 1, 2, 3). We found that aerobic metabolism was reduced in the sCLU and S100B cell lines, and that synaptic signaling pathways were reduced in S100B-transfected cells, at normoxic conditions. These transcriptomic responses might reduce production of reactive oxygen species (ROS), while also reducing the energy consumption of neuronal cells. Additionally, autophagy processes appeared to be important for cellular homeostasis in sCLU-transfected cells, which might ultimately protect cells from apoptosis. When oxidatively stressed, sCLU- and S100B-transfected cells did not mount similar gene regulatory responses, but nevertheless demonstrated improved viability compared to mock-transfected cells, presumably due to a pre-adaptive (constitutional) response, seen already under normoxic conditions, in preparation for upcoming stress conditions. In contrast to this effect, the nCLU cell line exhibited elevated stress and apoptosis pathways in response to oxidative stress, which suggests a reduced basal protection against oxidative damage in this cell line. Furthermore, known hypoxia response genes and pathways, such as HIF1A signaling and glycogen metabolism, were enhanced in transfected cells when exposed to hypoxic conditions. While the roles of CLU and S100B in neurodegenerative diseases are being debated, we found evidence for the upregulation of neuroprotective effects in cell lines overexpressing these genes, in response to hypoxia and oxidative stress.

**Table 1:**
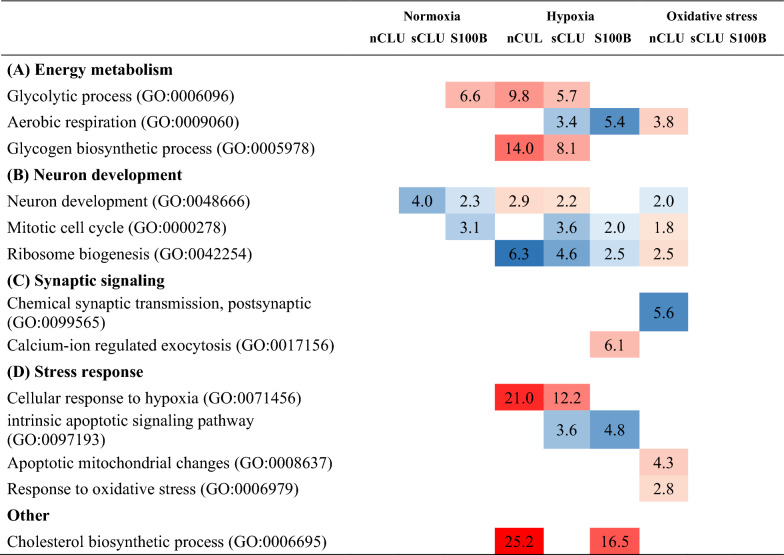
Selection of significant GO terms enriched in transfected cell lines compared to mock cell line

Significant GO terms related to (A) energy metabolism, (B) neuron development, (C) synaptic signaling and (D) stress response, were selected that were enriched in upregulated (red) and downregulated genes (blue) for each cell line and treatment, when compared to the mock cell line at each respective condition. Displayed are fold enrichments of pathways

**Table 2:**
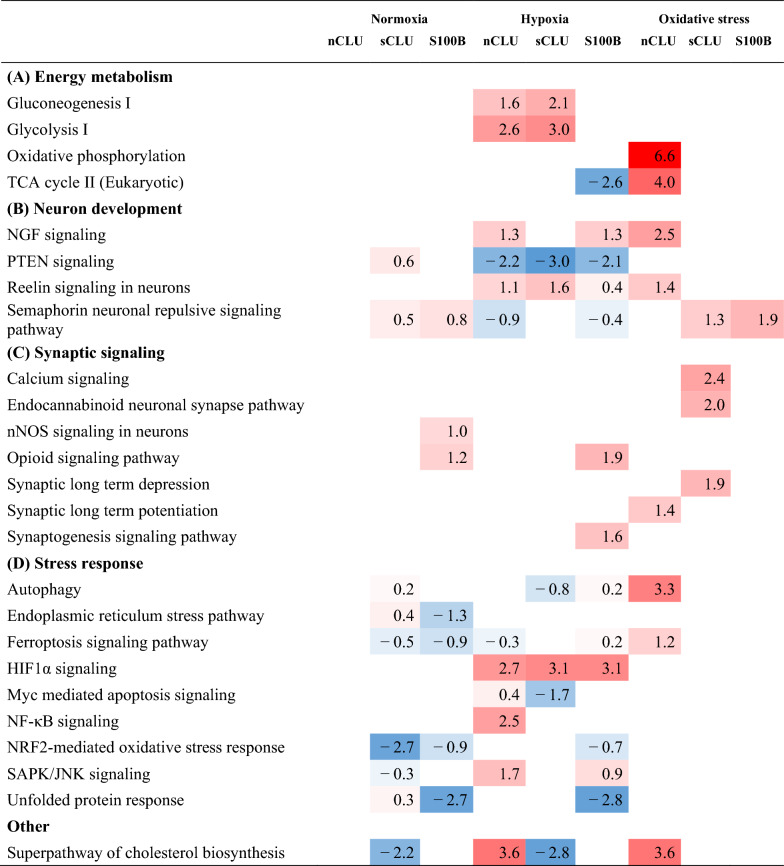
Selection of significant IPA canonical pathways for transfected cell lines compared to mock cell line

Significant IPA canonical pathways related to (A) energy metabolism, (B) neuron development, (C) synaptic signaling and (D) stress response. Activation/inhibition of pathways are indicated by positive (red)/negative (blue) IPA z-score values

**Table 3:**
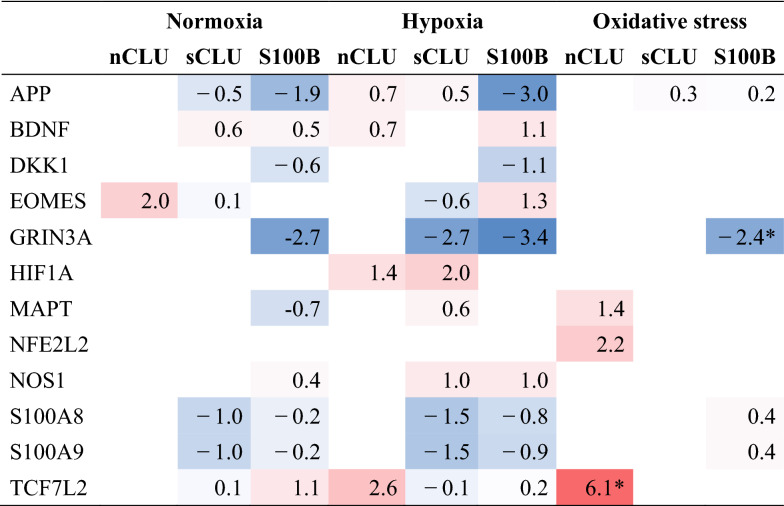
Selection of interesting upstream regulators for transfected cell lines, when compared to mock cell line

Interesting upstream regulators were selected as ‘activated’ (red)/‘inhibited’ (blue) in comparison to mock cell line, by use of the IPA Activation z-score. *Regulation bias, implying that X-fold change of upstream regulator does not match expected regulation of targets in its network

The findings of the present study have been demonstrated in a cell culture model and effects would still need to be confirmed in vivo. Unfortunately, it is not feasible to obtain samples from naturally diving hooded seals that experience hypoxia. Alternatively, fresh brain slices exposed to hypoxia in vitro could mimic more closely natural conditions than cell culture. However, capturing hooded seals and performing experiments on fresh tissue requires great effort and have only been done on rare occasions [14, 39]. Cell culture experiments therefore represent a great possibility to mimic hypoxic conditions.

In this study, we highlighted pathways and targets of hypoxia tolerance that may provide clues to tackle neurodegenerative diseases such as Alzheimer’s disease and Parkinson’s disease. While the cell culture experiments indicated neuroprotective effects of CLU and S100B at hypoxia and oxidative stress, these results yet require confirmation in in vivo studies.

## Methods

### Cell culture

HN33 cells (murine hippocampal neurons × neuroblastoma) [60] (American Type Culture Collection, Rockville, MO) had been stably transfected with each candidate gene (nuclear clusterin (nCLU), soluble clusterin (sCLU) and S100B) and an empty vector (mock), respectively [31]. The four cell lines were cultivated in Dulbecco’s Modified Eagle Medium (DMEM) (Biowest, Darmstadt) containing 10% fetal calf serum (FCS) (Biowest, Darmstadt, Germany) and 1% of a mixture of penicillin and streptomycin (PAA, Pasching, Austria) at 37 °C in a humidified atmosphere and 5% CO_2_. The medium of the transfected cells was supplemented with 700 µg/ml geniticin (PAA, Pasching, Austria).

### Quantitative real-time PCR

The successful overexpression of target genes was verified before and after experiments by qRT-PCR as described in Geßner et al. [31]. For that purpose, RNA was extracted from cells using the Crystal RNA Mini Kit (BiolabProducts, Gödenstorf, Germany) including an on-column DNA digestion with RNase-free DNase (Qiagen, Germany). First-strand cDNA was synthesized from 1 µg of isolated RNA with Oligo(dT)_18_ primer using the Fermentas RevertAid H Minus First Strand cDNA Synthesis Kit (Thermo Scientific, Schwerte, Germany). The qPCR was performed with a 7500 Fast Real-Time PCR System and the Power SYBR Green master mix (Applied Biosystems, Darmstadt, Germany) using a standard PCR protocol (step 1–2: 50 °C 2 min, 95 °C 10 min; 40 cycles step 3–5: 95 °C for 15 s, 60 °C for 30 s, 72 °C for 30 s) including melting curve analysis. Absolute mRNA copies were calculated with the 7500 System Sequence Detection Software 2.0.6 (Applied Biosystems) using recombinant plasmid dilutions of 10^2^–10^8^ as standard curve, and then normalized to 1 µg of total RNA.

### Normoxia, hypoxia and oxidative stress treatment

Experiments were conducted in 96-well plates containing 3.75 × 10^4^ cells per well diluted in 50 µl DMEM medium (10% FCS, 1% Penicillin/Streptomycin) of each transfected cell line at passage 38, including a cell line transfected with an empty vector (mock cell line). Cells were exposed to normoxia (21% O_2_, 5% CO_2_, 37 °C), hypoxia (1.2% O_2_, 5% CO_2_, 93.8% N2, 37 °C) and oxidative stress (275 µM H_2_O_2_ in 50 µl DMEM per well, 21% O_2_, 5% CO_2_, 37 °C) for 24 h, respectively. After trypsinization every 6 wells were pooled and used as one replicate, generating four replicates per cell line and treatment. Samples were centrifuged at 180×*g* for 5 min, supernatant removed and pellets stored at − 20 °C until further processing.

We note that we considered 21% as normoxic condition. The HN33-cells used in this study were cultured at 21% O_2_ since their dissociation and somatic cell fusion with neuroblastoma cells [60]. In these conditions, the cells displayed expression of neurofilaments and electrophysiological behavior typical of hippocampal neurons [60]. While other hippocampal neurons may experience 21% O_2_ as hyperoxic, the HN33-cells have been exposed to 21% O_2_ over many generations and hence arguably perceive this condition as normoxic. Related studies (e.g. [31, 53, 54]) also considered 21% as normoxia.

### Cell viability assay

Cell viability was assessed by CellTiter-Glo® (CTG) Luminescent Cell Viability Assay Kit (Promega, Mannheim, Germany) according to the manufacturer’s instructions. The assay determines the ATP content of the cells and serves as reliable indicator of the number of healthy, metabolically active cells [76]. After incubation at normoxia, hypoxia and oxidative stress, as mentioned above, CTG reagent was added and luminescence measured by a DTX 880 Multimode Detector (Beckmann Coulter, Krefeld, Germany). Statistical analysis was conducted in R version 4.1.2 [85]. Robust triplicates were determined and intensities normalized to the mock cell line at normoxic conditions. Pairwise t-test and false discovery rate (FDR) multiple correction testing was performed using the *compare_means* function of ggpubr package, with the mock cell line as reference group at each respective condition [49].

### RNA preparation and RNA-Seq

Total RNA of frozen cell pellets was extracted with the Crystal RNA Mini Kit (BiolabProducts, Gödenstorf, Germany) after the manufacturer’s instructions, including an on-column DNA digestion with RNase-free DNase (Qiagen, Germany). RNA integrity and quantity were assessed with the Agilent 4200 TapeStation system (Agilent Technology, Sanat Clara, USA) and triplicates determined for sequencing. The cDNA libraries were generated with 500 ng of total RNA after rRNA depletion, and sequenced on a NovaSeq platform with a setting of 150 bp paired-end reads and an estimated output of 50 million reads (GeneWiz, Leipzig, Germany). The raw transcriptome files are available from the NCBI Sequence Read Archive (SRA) from cell lines transfected with mock vector, nCLU, sCLU and S100B at normoxic, hypoxic and oxidative stress conditions (Additional file 1: Table S1).

### Transcriptome analysis

Sequencing files were uploaded to a Galaxy platform in fastq.gz format for further analysis. A sequencing quality report was generated using FastQC (Galaxy Version 0.72) and MultiQC (Galaxy Version 1.7). Since read quality was good (average Phred score > 35, Additional file 1: Table S1), no further read trimming was performed. Reads were mapped against the mouse reference genome GRCm39 (http://www.ncbi.nlm.nih.gov/assembly/GCF_000001635.27/, genomic FASTA and GTF) with Bowtie2 (Galaxy Version 2.3.4.2) with the very sensitive end-to-end preset setting (–very-sensitive). Mapped reads from generated genome BAM files were filtered by a minimum mapping quality of 10 and determined with featureCounts (Galaxy Version 1.6.3 + galaxy2), counting aligned fragments (even when only one paired read mapped) and excluding chimeric fragments. Reads were allowed to contribute to one feature only. Differentially expressed genes (DEGs) were determined from count tables using DESeq2 (Galaxy Version 2.11.40.6), performing pairwise comparisons with the mock cell line at the respective condition as reference. Only genes with a corrected FDR p-value < 0.05 were considered significant. Principal component analysis (PCA) was performed on count tables from all cell lines and treatments with DESeq2 (Version 1.32.0) in R (Version 4.1.0). Gene Ontology (GO) analysis was performed using PANTHER Overrepresentation Test (Protein Analysis Through Evolutionary Relationships, http://go.pantherdb.org/, GO Ontology database 10.5281/Zenodo.5228828 Released 2021-08-18) [70]. The annotated mouse genes in the PANTHER DB were used as a reference list, and overrepresentation was tested in GO and GO Slim terms and Reactome pathways with Fisher's Exact Test with FDR multiple testing correction. Only categories with corrected p-values < 0.05 were considered significant. Enrichment in Canonical Pathways and Upstream Regulator Analysis were performed with Qiagen’s Ingenuity Pathway Analysis (IPA, Qiagen, Hilden, Germany) Core analysis tool.

## Supplementary Information

**Additional file 1: Figure S1.** Expression of endogenic and transgenic CLU and S100B sequences in transfected HN33 cell lines at normoxia, determined by qPCR experiments. Differences in Ct-values between endogenic and transgenic nCLU, sCLU and S100B were 13.17 (with Ct of 40 for endogenic nCLU), 13.25 and 9.48, respectively. The fold-expression difference for nCLU, sCLU and S100B therefore were 2^13.17^, 2^13.25^ and 2^9.48^, respectively. **Figure S2.** TPM values of endogenic (CLU, S100B) and transgenic [CLU (Ccr), S100B (Ccr)] sequences in transfected cell lines (mock, nCLU, sCLU, S100B) at normoxia, hypoxia and oxidative stress. **Table S1.** Sequencing and mapping overview. Triplicates were sequenced per cell line and oxygen treatment. For replicate mock-H_2_O_2_-2 sequencing failed and was discarded. Around 51 million reads per sample were generated of which around 75% mapped to the GRCm39 mouse reference genome.

## Data Availability

Sequence data that support the findings of this study have been deposited in the Sequence Read Archive (SRA) with the primary accession code PRJNA86312, available at: https://www.ncbi.nlm.nih.gov/sra/?term=PRJNA862312.
