# Supplementary material for: Transcriptomes of Clusterin- and S100B-transfected neuronal cells elucidate protective mechanisms against hypoxia and oxidative stress in the hooded seal (Cystophora cristata) brain
Source: BMC Neurosci. 2022 Oct 15;23:59. doi: 10.1186/s12868-022-00744-6 (PMC9571494; doi:10.1186/s12868-022-00744-6)
Supplement: Supplementary file 1 — Additional file 1: Figure S1. Expression of endogenic and transgenic CLU and S100B sequences in transfected HN33 cell lines at normoxia, determined by qPCR experiments. Differences in Ct-values between endogenic and transgenic nCLU, sCLU and S100B were 13.17 (with Ct of 40 for endogenic nCLU), 13.25 and 9.48, respectively. The fold-expression difference for nCLU, sCLU and S100B therefore were 213.17, 213.25 and 29.48, respectively. Figure S2. TPM values of endogenic (CLU, S100B) and transgenic [CLU (Ccr), S100B (Ccr)] sequences in transfected cell lines (mock, nCLU, sCLU, S100B) at normoxia, hypoxia and oxidative stress. Table S1. Sequencing and mapping overview. Triplicates were sequenced per cell line and oxygen treatment. For replicate mock-H2O2-2 sequencing failed and was discarded. Around 51 million reads per sample were generated of which around 75% mapped to the GRCm39 mouse reference genome. [file 12868_2022_744_MOESM1_ESM.docx]

**Transcriptomes of Clusterin- and S100B-transfected neuronal cells elucidate protective mechanisms against hypoxia and oxidative stress in the hooded seal (*Cystophora cristata*) brain**

Gerrit A. Martens^1^*, Cornelia Geßner^1^, Carina Osterhof^2^, Thomas Hankeln^2^, Thorsten Burmester^1^

^1^ Institute of Animal Cell and Systems Biology, Biocenter Grindel, University of Hamburg, 20146 Hamburg, Germany, ^2^ Institute of Organismic and Molecular Evolution, Molecular Genetics & Genome Analysis, Johannes Gutenberg University Mainz, 55128 Mainz, Germany, *corresponding author

**Additional figures**


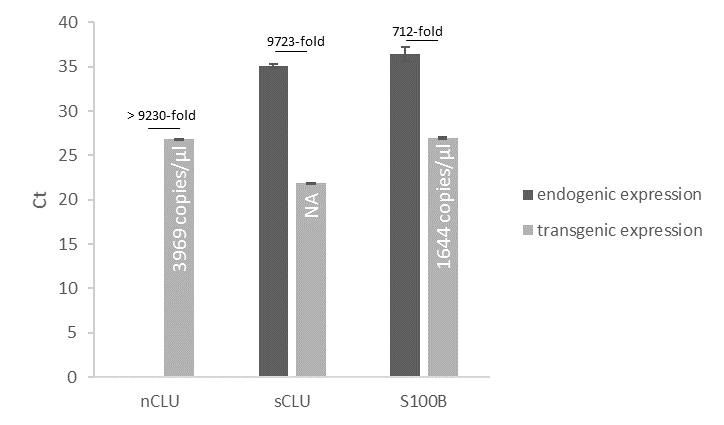


**Fig. S1**: Expression of endogenic and transgenic CLU and S100B sequences in transfected HN33 cell lines at normoxia, determined by qPCR experiments. Differences in Ct-values between endogenic and transgenic nCLU, sCLU and S100B were 13.17 (with Ct of 40 for endogenic nCLU), 13.25 and 9.48, respectively. The fold-expression difference for nCLU, sCLU and S100B therefore were 2^13.17^, 2^13.25^ and 2^9.48^, respectively.


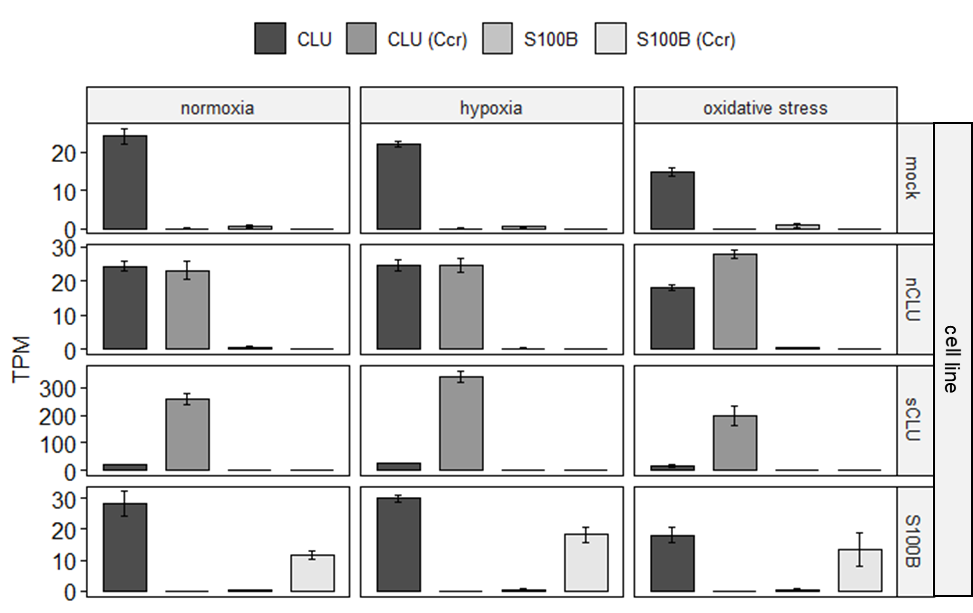


**Fig. S2**: TPM values of endogenic (CLU, S100B) and transgenic (CLU (Ccr), S100B (Ccr)) sequences in transfected cell lines (mock, nCLU, sCLU, S100B) at normoxia, hypoxia and oxidative stress

**Additional tables**

**Table S1**: Sequencing and mapping overview. Triplicates were sequenced per cell line and oxygen treatment. For replicate mock-H_2_O_2_-2 sequencing failed and was discarded. Around 51 million reads per sample were generated of which around 75 % mapped to the GRCm39 mouse reference genome.

| Sample ID | Accession | # Reads | Mean Quality Score | Mapped reads % |
| --- | --- | --- | --- | --- |
| HN33-mock-normoxia-1 | SRR20646231 | 54,631,566 | 35.93 | 75.93% |
| HN33-mock-normoxia-2 | SRR20646230 | 43,220,304 | 35.81 | 74.81% |
| HN33-mock-normoxia-3 | SRR20646219 | 51,775,704 | 36.00 | 78.57% |
| HN33-mock-hypoxia-1 | SRR20646208 | 52,052,511 | 35.83 | 73.91% |
| HN33-mock-hypoxia-2 | SRR20646202 | 58,723,186 | 35.81 | 76.67% |
| HN33-mock-hypoxia-3 | SRR20646201 | 46,880,076 | 35.89 | 75.66% |
| HN33-mock-oxidative_stress-1 | SRR20646200 | 30,508,905 | 35.97 | 74.34% |
| HN33-mock-oxidative_stress-2 | SRR20646199 | 40,445,197 | 35.97 | 76.49% |
| HN33-nCLU-normoxia-1 | SRR20646198 | 65,965,761 | 35.89 | 77.78% |
| HN33-nCLU-normoxia-2 | SRR20646197 | 48,021,315 | 35.96 | 79.58% |
| HN33-nCLU-normoxia-3 | SRR20646229 | 47,677,448 | 35.90 | 74.37% |
| HN33-nCLU-hypoxia-1 | SRR20646228 | 58,624,401 | 35.87 | 72.79% |
| HN33-nCLU-hypoxia-2 | SRR20646227 | 51,146,599 | 35.87 | 77.06% |
| HN33-nCLU-hypoxia-3 | SRR20646226 | 48,122,292 | 35.96 | 77.99% |
| HN33-nCLU-oxidative_stress-1 | SRR20646225 | 55,650,122 | 35.81 | 74.18% |
| HN33-nCLU-oxidative_stress-2 | SRR20646224 | 43,358,527 | 35.75 | 70.73% |
| HN33-nCLU-oxidative_stress-3 | SRR20646223 | 63,395,372 | 35.79 | 74.16% |
| HN33-sCLU-normoxia-1 | SRR20646222 | 49,471,413 | 35.79 | 76.94% |
| HN33-sCLU-normoxia-2 | SRR20646221 | 52,628,491 | 35.75 | 69.68% |
| HN33-sCLU-normoxia-3 | SRR20646220 | 63,415,123 | 35.84 | 75.52% |
| HN33-sCLU-hypoxia-1 | SRR20646218 | 52,775,071 | 35.86 | 75.87% |
| HN33-sCLU-hypoxia-2 | SRR20646217 | 43,037,551 | 35.84 | 77.59% |
| HN33-sCLU-hypoxia-3 | SRR20646216 | 40,234,863 | 35.95 | 80.70% |
| HN33-sCLU-oxidative_stress-1 | SRR20646215 | 27,019,498 | 35.51 | 59.72% |
| HN33-sCLU-oxidative_stress-2 | SRR20646214 | 54,622,362 | 35.85 | 75.17% |
| HN33-sCLU-oxidative_stress-3 | SRR20646213 | 53,908,929 | 35.73 | 74.65% |
| HN33-S100B-normoxia-1 | SRR20646212 | 53,322,771 | 35.81 | 76.70% |
| HN33-S100B-normoxia-2 | SRR20646211 | 62,184,734 | 35.84 | 76.67% |
| HN33-S100B-normoxia-3 | SRR20646210 | 58,811,703 | 35.97 | 79.11% |
| HN33-S100B-hypoxia-1 | SRR20646209 | 55,875,817 | 35.81 | 76.60% |
| HN33-S100B-hypoxia-2 | SRR20646207 | 40,033,728 | 35.95 | 78.91% |
| HN33-S100B-hypoxia-3 | SRR20646206 | 43,408,060 | 35.92 | 79.04% |
| HN33-S100B-oxidative_stress-1 | SRR20646205 | 46,397,569 | 35.90 | 72.36% |
| HN33-S100B-oxidative_stress-2 | SRR20646204 | 47,776,179 | 35.97 | 75.28% |
| HN33-S100B-oxidative_stress-3 | SRR20646203 | 82,370,114 | 35.78 | 74.25% |
